# Hydrophobic Silica Gels and Aerogels for Direct Air Capture: Hybrid Grafting To Suppress Water Uptake and Capillary Condensation

**DOI:** 10.1021/acsami.5c04491

**Published:** 2025-07-17

**Authors:** Paweł P. Ziemiański, Mercy Cherotich, Ilia Sadykov, Chaima Mansour, Ivan Lunati, Marco Ranocchiari, Wim J. Malfait, Sandra Galmarini

**Affiliations:** 1 Building Energy Materials and Components, Empa, 8600 Dübendorf, Switzerland; 2 Laboratory for Computational Engineering, Empa, 8600 Dübendorf, Switzerland; 3 PSI Center for Energy and Environmental Sciences (CEE), 5232 Villigen PSI, Switzerland

**Keywords:** direct air capture, amine-functionalized sorbents, superhydrophobic, silica aerogel, CO_2_/water selectivity, surface modification, hybrid grafting functionalization

## Abstract

Direct air capture of CO_2_ (DAC) is critical to achieving climate goals, requiring the removal of approximately 220 000 Mt of CO_2_ by 2100. Amine-functionalized sorbents are promising for CO_2_ capture but face challenges under high humidity, where water sorption reduces the CO_2_ selectivity and capillary condensation may degrade the sorbent, especially the delicate silica aerogel backbone, compromising structural integrity. To address this issue, we introduce superhydrophobic silica gels functionalized by hybrid grafting of amine, long-aliphatic, and trimethylsilyl groups. These materials drastically reduce water uptake, from >50 wt % for nonhydrophobized gels to 5 wt % at 95% RH, and maintain high CO_2_/water selectivity at high relative humidity. This shows how to overcome a limitation of silica sorbents, which have shown poor performance up to now under realistic high-humidity DAC conditions. The hydrophobic functional groups shield the hydrophilic amine sites and prevent capillary condensation, preserving pore hydrophobicity, preventing pore clogging with water, and ensuring rapid CO_2_ diffusion, essential for sustained performance. We demonstrate that conventional single-component and dry-condition CO_2_ sorption tests overlook the critical role of moisture, underestimating the detrimental effect of presorbed water on nonhydrophobized silica gels, previously reported in the literature. The ability to apply ambient pressure drying without significant selectivity loss, along with the affordability of the silica matrix, enhances the feasibility of real-world implementation for silica-based DAC sorbents. Given the versatility of amorphous sorbents, this study paves the way for further material optimization tailored to DAC applications.

## Graphical abstract

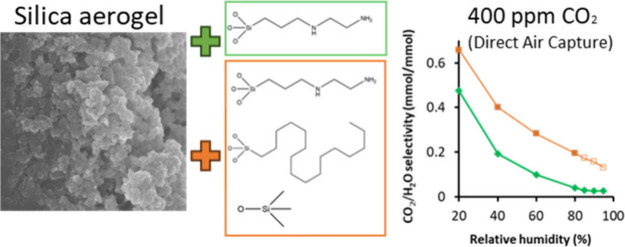

## 1 Introduction

More than 99.9% of the studies published in peer-reviewed journals agree that human activity is the leading cause of ongoing climate change.^1^ Carbon dioxide (CO_2_) is the primary and longest-lived greenhouse gas released into the atmosphere,^2^ and its concentration is still rising, reaching an average of 417.9 ± 0.2 ppm in 2022 ^3^ and 422.1 ppm in 2024.^4^ The increase in CO_2_ concentration is related to fossil fuel combustion, cement manufacturing, and change in land use (primarily deforestation).^5^ Capture of CO_2_ has proven effective in maintaining safe levels of CO_2_ aboard spacecraft and submarines, environments where this could not be achieved by ventilation.^6^ Hence, direct air capture (DAC) offers a potential mitigation pathway to counterbalance the hard-to-abate residual greenhouse gas emissions.^7^

In order to limit global warming to 1.5 °C with immediate action and limited overshoot, a cumulative negative emission of approximately 220 000 Mt of CO_2_ by 2100 is required.^8^ By 2022, there were 19 DAC plants operating worldwide, capturing only 0.01 Mt of CO_2_/year.^9^ The estimated selling cost of negative emission of 1 t of CO_2_ by 2022 was $1200, which is expected to drop to $600–1000 by 2030,^10^ while the average total cost of carbon emission (TCE) remains low (e.g., TCE for transport sector was $110 in 2021).^11^ It is critical to focus on reducing operational costs and enhancing sorbent efficiency to ensure the economic viability of the DAC.

Energy requirements, which are critical components of DAC operating costs, are heavily influenced by the sorption properties of the material. Selectivity toward CO_2_ remains a key parameter due to the high enthalpy of H_2_O desorption, which is in the range of its evaporation.^12–14^ The mean monthly relative humidity (RH) over most of Europe is between 70 and 95%^15^ and periodically reaches 100% when fog is present. For example, such a high RH corresponds to 22.2 mbar for 70% relative humidity at 25 °C and 11.0 mbar for 90% RH at 10 °C. At the same time, the CO_2_ concentration is highly diluted (∼0.4 mbar of partial pressure). Under such conditions, CO_2_ cannot compete with H_2_O for active sites in common physical sorbents.^16^

Amine-functionalized sorbents have shown great promise due to their strong affinity for CO_2_ through the chemisorption pathway.^14,17–19^ Most commonly, amino-functionalized sorbents are prepared either by physical impregnation with polymeric amine species (e.g., tetraethylenepentamine or polyethylenimine) or by chemical grafting of amino groups to support surfaces. However, despite relatively higher CO_2_ adsorption capacities, physically impregnated sorbents suffer from poor thermal and water stability and significantly lose their performance with each cycle.^20–24^

In contrast to physisorbents, amine-functionalized sorbents can not only retain CO_2_ adsorption under humidity, but also CO_2_ sorption can be possibly enhanced due to different reaction mechanisms.^25,26^ Without water, one molecule of CO_2_ is adsorbed by two primary or secondary amino groups with the formation of ammonium carbamate:

$$2{\mathrm{R‐NH}}_{2}+{\mathrm{CO}}_{2}⇌{\mathrm{R‐NH}}_{2}{\mathrm{‐COO}}^{-}+{{\mathrm{R‐NH}}_{3}}^{+}$$

When water is present, primary, secondary, and tertiary amines can sorb one molecule of CO_2_ per amino group with the formation of bicarbonate:

$${\mathrm{R‐NH}}_{2}+{\mathrm{CO}}_{2}+H_{2}O⇌{{\mathrm{R‐NH}}_{3}}^{+}{{\mathrm{HCO}}_{3}}^{-}$$

However, high water concentration close to saturation pressure may result in significantly higher H_2_O than CO_2_ uptake due to the physical adsorption of water on the available surfaces and capillary condensation.^13,19,27,28^ Excessive water uptake reduces the energetic efficiency of DAC and leads to H_2_O adsorption and condensation that might block pores, hindering the transport of CO_2_ through the porous structure of the sorbent.

To date, most of the studies on DAC adsorbents have not reported CO_2_ sorption under humid conditions (close to 80–90% of RH) or quantified water sorption under realistic operational conditions.^14,17,18^ Therefore, a direct comparison of most potential DAC sorbents under realistic conditions is not possible. On the other hand, coadsorbed water, released during the desorption stage, can lower the CO_2_ partial pressure, effectively increasing the cycled CO_2_ mass. However, for the sorbent studied by Wang and Li,^12^ the water-promoted CO_2_ desorption is only energetically favorable under low and unrealistic RH (∼30%), while the quantity of water adsorbed at higher RH significantly increased energy consumption.

Silica aerogels have porosity predominantly consisting of mesopores (2–50 nm)^29^ and large specific surface area (300–1200 m^2^/g).^30,31^ The skeleton of the silica aerogels consists of fundamental silica nanoparticles (diameter 2.5–5 nm) linked by the interparticle necks into a percolated 3D network with tailorable porosity.^32^ The ease of pore structure and surface chemistry tailoring indicates that silica gels may offer advantages over ordered mesoporous silica. The surface of silica nanoparticles is terminated with silanol and/or alkoxy groups, which can be further replaced during grafting.^31,32^ While grafting of amino groups allows for CO_2_ chemisorption, termination of silica particles with hydrophobic trimethylsilyl groups not only decreases water adsorption but also allows for much cheaper ambient pressure drying of gels (xerogels), in contrast to the more expensive supercritical drying method (aerogels).^32^ Other hydrophobic groups, like long aliphatic hexadecyl chains, were also shown to greatly improve silica hydrophobicity.^33^

Several studies have explored the potential of amine-grafted silica materials for CO_2_ capture, with particular attention paid to ordered mesoporous silica, including MCM and SBA families.^14,34,35^ While the functionalization of silica gels in the context of CO_2_ capture has been less extensively studied, several research^36–41^ and review studies^42,43^ have offered valuable insights into this area.

Wörmeyer and Smirnova^37^ found a positive correlation between amine loading in silica gels and CO_2_ adsorption capacity. However, higher amine loading was negatively correlated with specific surface area. The authors exposed the samples to low relative humidity (30%) during the CO_2_ sorption, which modestly enhanced the CO_2_ capacity of the samples with grafted triamino groups. All tested samples sorbed ∼2 times more H_2_O than CO_2_ sorption (mol/mol) under 2500 ppm of CO_2_ (∼6 times the CO_2_ concentration of ambient air) and 30% RH. Wörmeyer et al.^36^ compared amine grafting methods, finding that co-condensation allowed higher amine loading but failed at enhancing CO_2_ sorption at low pressures (2500 ppm). In contrast, the samples functionalized after gelation sorbed up to 0.5 mmol of CO_2_/g at 2500 ppm of CO_2_. Wörmeyer and Smirnova^41^ investigated the kinetic characteristics of CO_2_ sorption under 30% RH on silica gels with grafted mono and triamine. The calculated pore diffusion coefficients were on the order of 3.5 × 10^–6^ m^2^/s. However, the authors were unable to fit the shape of the breakthrough curve properly, making the obtained coefficient questionable.

Jiang et al.^40^ developed spherical amino-functionalized silica gels with amines using low-cost vacuum drying. The samples showed significant sorption of CO_2_ from 1% vol gas stream and good cyclability. No water was used during the sorption study. Cui et al.,^38^ showed that water vapor enhances CO_2_ uptake in amine-modified silica aerogels, but high water content eventually hinders performance in CO_2_ adsorption. However, the authors used a 250 times more concentrated CO_2_ stream than that in ambient air. Begag et al.^39^ developed amine-functionalized aerogel with a hydrophobic solid framework by condensation of methyltrimethoxysilane. However, the water uptakes on these sorbents are not shown. Moreover, the H_2_O was only used during sorbent regeneration. As presented above, silica gels are potentially promising sorbents for direct air capture. To summarize, while existing data indicate that water uptake at high relative humidities poses a limitation to the use of silica aerogel for DAC, data comparing the CO_2_ and H_2_O sorption behavior under realistic high relative humidity and low concentrations of CO_2_ (∼400 ppm) are nonexistent.

In the present study, we introduce a novel class of superhydrophobic silica-gel-based sorbents that are pushing the limits of the CO_2_/H_2_O selectivity of silica-based sorbents functionalized with amines, which, without targeted hydrophobization, are inherently hydrophilic. The silica gels feature hybrid grafting of amino groups, superhydrophobic long aliphatic chains, and trimethylsilyl groups. We present an in-depth analysis of their CO_2_ and H_2_O cosorption behaviors under realistic high relative humidity relevant for direct air capture of CO_2_. In this work, we systematically explore how surface functionalization and drying methods influence the sorbent performance. We demonstrate that silica gels functionalized by amine grafting, but without the additional hydrophobization, are not suitable for DAC of CO_2_ under realistic conditions due to their higher affinity for water. We compare the selectivity of our tailored sorbents to that of the benchmark amine-functionalized resin sorbent. Our findings highlight the potential of amine-grafted silica gel as a cost-effective and CO_2_-selective solution for carbon capture.

## 2 Experimental Section

### 2.1 Sample Synthesis

The silica gel samples were synthesized using the sol–gel method using a partially prehydrolyzed ethyl silicate Dynasylan 40 (Evonik) as a silica precursor with an equivalent silicon dioxide content of 40%. This silica precursor was diluted with anhydrous ethanol (EtOH) to achieve 20 wt % concentration of silica in EtOH. 0.157 g of H_2_O and 3.2 × 10^–4^ mL of 70% wt HNO_3_ per 1 g of Dynasylan 40 were added to advance silica hydrolysis and condensation and to stabilize the sol (P750).^44^ The sol was then left for 24 h and used the next day.

This prehydrolyzed sol was used in a classical two-step, acid–base catalyzed sol–gel process adapted from ref 31. The sol was mixed with H_2_O (1 vol. sol:0.1 vol H_2_O), and gelation was triggered by adding 5 × 10^–3^ ml of 5.5 M NH_3_·H_2_O per 1 mL of sol. Obtained alcogels were aged for 20 h at 65 °C to improve their mechanical properties. Gel samples of lower densities (and wider porosity) were obtained by mixing the sol with EtOH with 1:1 or 1:2 sol to EtOH proportions and adjusting the added NH_3_·H_2_O quantity to keep the volumetric concentration constant. The silica alcogel samples were washed with EtOH and put into *X*% wt solution of (*N*-3-trimethoxysilyl)­propylethylenediamine (2amino-Si­(OMe)_3_) DAPS; Sigma-Aldrich) or *N*′-(3-trimethoxysilylpropyl)­diethylenetriamine (TAMS; Sigma-Aldrich) in EtOH with the proportion of 1 vol of alcogel to 1.5 vol functionalizing solution, similar to the method proposed in ref 36. The samples were kept sealed at 65 °C for 20 h. The hydrophobization was obtained by a sequential treatment of amine-functionalized samples with 10% wt hexadecyltrimethoxysilane (16C-Si­(OMe)_3_; Sigma-Aldrich) in EtOH and 6% wt hexamethyldisilazane (HMDS), both in EtOH, following precisely the amine-grafting method described above. The samples were washed with EtOH and then dried in supercritical CO_2_ in an automated autoclave-pump Separex setup (SCD, 50 °C, 120 bar, 10 h) or under a flow of dry nitrogen at 150 °C for 2 h (ambient pressure). The scheme of gel synthesis and postfunctionalization, as well as the morphology of the samples after ambient and supercritical drying, are shown in Figure 1.

**Figure 1:**
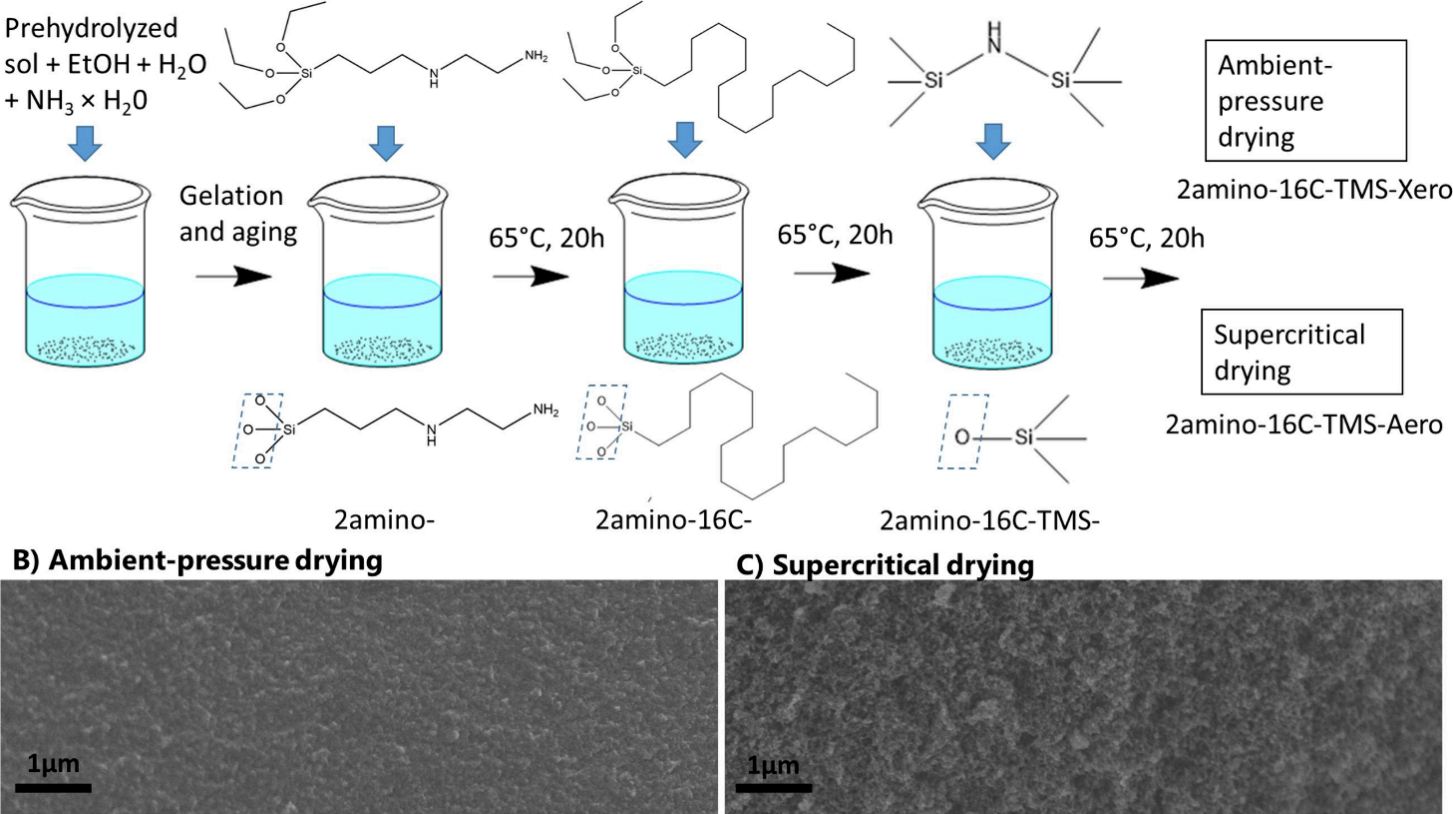
(A) General scheme of gel synthesis and functionalization. Scanning electron microscopy images on (B) xerogel (2amino-16C-TMS-3Vol-Xero) and (C) aerogel (2amino-16C-TMS-3Vol-Aero). Details of the imaging can be found in the Supporting Information.

Depending on the functionalization, hydrophobization, addition of ethanol to lower the gel density, and drying methods, the samples are named as follows: 2amino-16C-TMS-Aero, where 2amino stands for grafted 2amino-Si­(OMe)_3_ (3amino in case of TAMS grafting), 16C corresponds to the grafted hexadecyl aliphatic chain (16C-Si­(OMe)­3), TMS refers to post-treatment with HDMS, which resulted in grafting of trimethylsilyl groups (TMS), and Aero indicate that the sample was dried in supercritical CO_2_ (aerogel; contrary to drying under ambient pressure indicated by Xero—xerogel). Suppose ethanol was added to the sol just before gelation to decrease the gel density. In that case, xVol. term is added, where x represents the proportional dilution of the sol with ethanol (e.g., 3Vol – 1 volume of sol mixed with 2 volumes of ethanol). 2xConc.- prefix refers to the twice as high a concentration of 2amino-Si­(OMe)_3_ used during amine-functionalization with respect to the other samples. For example, the 2amino-Xero sample was functionalized with 2amino-Si­(OMe)_3_, dried at ambient pressure, and without hydrophobization. 2amino-16C-TMS-3Vol-Aero—sample, whose density was lowered by addition of 2 vol of EtOH to 1 vol of p750 sol before gelation. The obtained alcogel was functionalized with 2amino-Si­(OMe)_3_, then hydrophobized by grafting 16C aliphatic chains and trimethylsilyl groups, and dried in supercritical CO_2_.

The silica gel samples synthesized in this study were compared to the commercial sorbent Lewatit VP OC 1065, a macroporous divinylbenzene cross-linked polymer in spherical bead form with primary amine groups.

### 2.2 Characterization Methods

#### 2.2.1 N_2_ and CO_2_ Adsorption (Single Component Volumetric Method)

Subcritical nitrogen adsorption measurements were performed using a 3Flex device (by Micromeritics). 50–80 mg of each sample was loaded into glass sample tubes and activated at the 3Flex degassing station under high vacuum (∼0.001 mbar) at 100 °C for 20 h. The adsorption and desorption isotherms were recorded at liquid nitrogen temperature (77.3 K) between 0.002 to 0.998 *p*/*p*_0_. Specific surface area BET (*S*_BET_; Brunauer, Emmett, and Teller)^45^ was calculated using Flex 6.02 software (by Micromeritics), following the fitting procedure proposed for materials containing micropores.^46^ The nitrogen molecular cross-sectional area used in the calculation was 0.162 nm^2^. Pore size distribution was calculated using the classical Barrett, Joyner, and Halenda (BJH) method,^47^ based on the Kelvin equation and the Harkins–Jura statistical thickness equation,^48^ assuming cylindrical pore geometry. The average pore diameter was also calculated from the total pore volume from the sorption isotherm (filling at 0.99 *p*/*p*_0_; *V*_total_N2_) and *S*_BET_ assuming cylindrical pores according to *d*_av_ = 4*V*_total_N2_/*S*_BET_, where *d* is the average pore diameter. Note that the total pore volume used here does not include possible macropores in the range, which is not probed by the nitrogen sorption analysis (≳100 nm). The CO_2_ adsorption isotherms were collected using ASAP 2020 (by Micromeritics) in the pressure range from 5 Pa to 1 bar. 100 mg of each sample was loaded into the sample tube and activated under high vacuum (∼5 μbar) at 100 °C for 20 h. During the experiments, the temperature was kept at 25.0 °C, thermostated by a CORIO CD-200F thermostat (by Julabo), with a stability of ±0.03 °C. In order to limit the experiment time, the equilibrium check interval was set to 45 s for pressures below 1 mbar, 30 s for pressures below 100 mbar, and 15 s for pressures higher than 100 mbar. This reduced the experimental time to 2 days per sample.

#### 2.2.2 Single-Component H_2_O Sorption

The water adsorption isotherms were measured using a VTI-SA+ vapor sorption analyzer (TA Instruments). 15–50 mg of samples was loaded onto a sample pan, then dried in situ at 100 °C under flow of pure nitrogen until stable mass was observed (<0.001 wt % change in 10 min). Then, water sorption and desorption isotherms were collected at 25 °C with a 5% RH step, up to 95%. The sample was assumed to be equilibrated when the mass change was <0.001 wt % per 10 min or a total step uptake time of 1440 min was reached.

#### 2.2.3 Elemental Composition Measurements

The carbon and nitrogen contents in the samples were measured by using a Unicube analyzer (by Elementar). Each sample was degassed at 80 °C for at least 12 h. Then, 1.000–2.000 mg of each sample were wrapped in aluminum foil and combusted in oxygen at 1450 °C. The wt % concentration of elemental carbon, hydrogen, and nitrogen was calculated by Unicube software, using conversion factors calibrated on sulfanilamide, prior to the analysis.

#### 2.2.4 Solid-State Nuclear Magnetic Resonance Spectroscopy

The nuclear magnetic resonance (NMR) spectra were collected on a Bruker spectrometer with a wide-bore 9.4 T magnet and the corresponding Larmor frequencies of 100.6 MHz for ^13^C and 79.5 MHz for ^29^Si. The samples were loaded in 7 mm zirconia holders with a magic angle spinning (MAS) rate of 4 kHz ± 2 Hz for measurements. For ^29^Si, nonquantitative ^1^H–^29^Si cross-polarization spectra were acquired with a contact time of 2000 μs and a pulse delay of 2 s (∼1.25 *T*_1_). For ^13^C, quantitative, single-pulse NMR spectra were collected with proton decoupling, with a 90° pulse of 8 μs, a 96 kHz spectral width, and 21 ms acquisition time. The recycle delay was set at 10 s, to ensure full relaxation (>5*T*_1_, a *T*_1_ relaxation time of 0.4 s was measured for the 2amino-16C-TMS-Aero sample). For ^13^C calibration, fully relaxed spectra were also collected for adamantine, octakis­(trimethylsiloxy)­silsesquioxane, and tris­(trimethylsilyl)­silane.^49^

#### 2.2.5 CO_2_–H_2_O Cosorption Breakthrough

The carbon dioxide sorption behavior under humid conditions was characterized with a BTA MK1 device from Surface Measurement Systems. The samples were carefully ground with an agate mortar and sieved to a size fraction between 0.2 to 0.4 mm (xerogels) or fragmented with razorblade (aerogels) to a size of approximately 0.5 mm. Then, samples were packed into 4 mm glass tubes, and the height of the adsorption bed was measured. The sample tube was weighted with and without the sample. Packed samples were then activated at 110 °C under a flow of dry nitrogen.

The whole experiment consisted of two adsorption cycles:

- The first adsorption cycle is presented in Figure 2A. After the sample activation, CO_2_ premixed in dry nitrogen at 400 ppm (1 bar) was diverted through the column (breakthrough phase; starting at time 0, Figure 2A). The second stage of this cycle consisted of a sequential increase in relative humidity from initially 0% to 20, 40, 60, and 80%. This was achieved by diverting part of the volumetric flow of 400 ppm of CO_2_ in nitrogen through a separate mass flow controller connected to a water saturator with deionized water. In this adsorption cycle, the adsorption of CO_2_ for each %RH step was recorded. After that, the sample was reactivated at 110 °C (stage not shown in Figure 2). During the reactivation step, the desorbed amount of CO_2_ was also recorded.
- The second cycle was carried out after sample reactivation (not presented in Figure 2B). The second cycle was used to evaluate CO_2_ sorption behavior in the already water-saturated sample. The sample was first saturated with 80% of RH without CO_2_ in this cycle. Then, a breakthrough of 400 ppm of CO_2_ in nitrogen with 80% RH was started (Figure 2B, time 0). After that, the sample was activated again, desorbed CO_2_ was quantified, and the dry mass of the sample was recorded.

**Figure 2:**
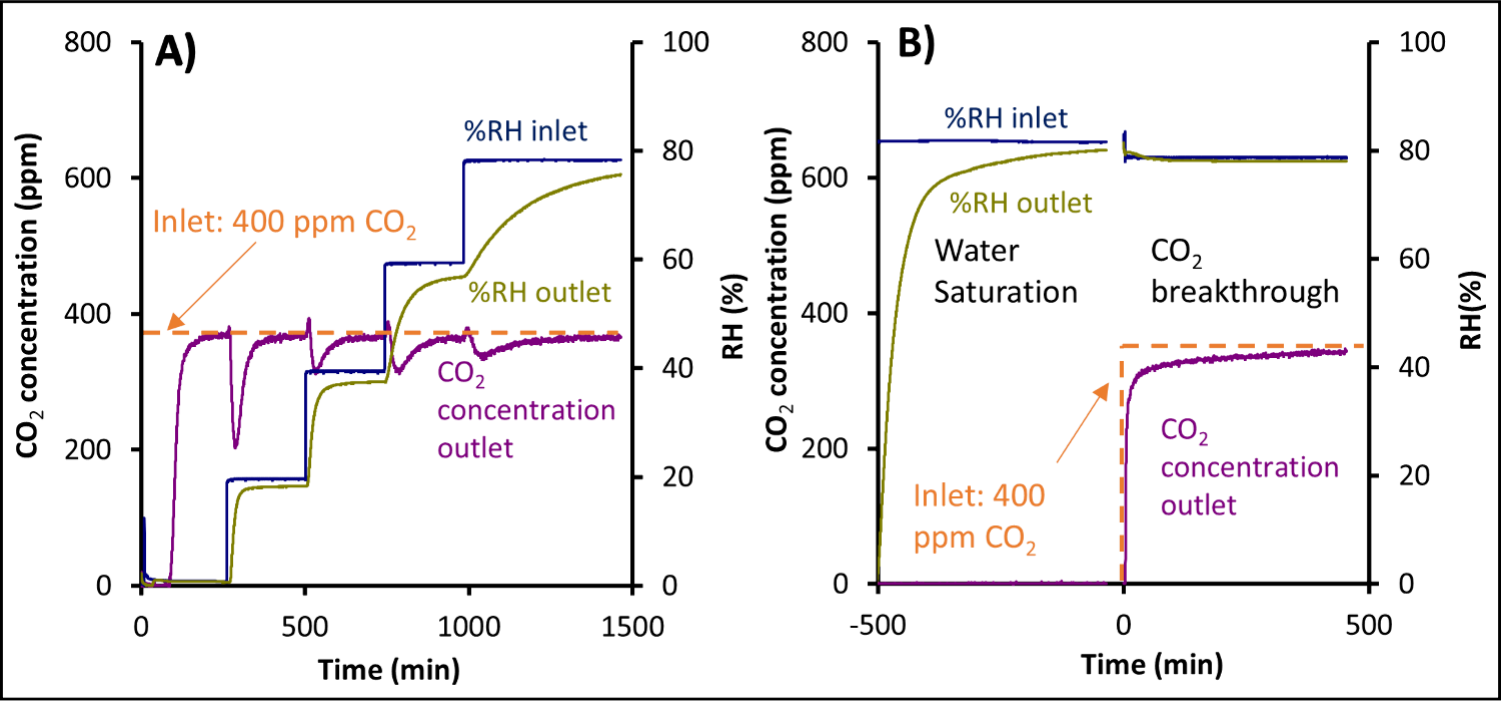
Breakthrough experiment on the 2amino-Xero sample using 400 ppm of CO_2_ in nitrogen. (A) First cycle: relative humidity is gradually increased from 0 to 80%. (B) Second cycle: the sample is first saturated with 80% of RH, and then the breakthrough with 400 ppm of CO_2_ starts. For clarity, sample (re)­activation, calibration stage, and blank experiments are not shown in the figure.

The CO_2_ concentration was measured only past the column with a Vaisala GMP252 probe (range 0–5000 ppm). Before each experiment, blank experiments were carried out, where the effects of dead volumes in the tubing system were accounted for from the measured profile of CO_2_ concentrations (not shown in Figure 2). A separate mass flow controller was used for the experimental steps with elevated RH. This “wet” mass flow controller was connected to the water saturator, with a headspace constantly saturated with 400 ppm of CO_2_, ensuring constant molar flow of CO_2_ when relative humidity was changed.

The RH was measured simultaneously at the inlet and outlet of the column with two Rotronic probes. The total flow was controlled with an Alicat M-200SCCM mass flow meter (250 sccm, ±0.6% reading accuracy). The CO_2_ concentration in the 400 PPM CO_2_ cylinder (PanGas) was precisely measured to be 391.70 ppm (±0.02 ppm) with the cavity ringdown spectroscopy technique using a Picarro G2401 analyzer (Empa, Switzerland). The CO_2_ concentration in the cylinder was measured over a 10 min period. The concentration was calculated against 4 traceable standards, measured before (2) and after (2) the gas sample. More details about the CO_2_ calibration scale can be found here:^50^

The adsorption of CO_2_ was calculated by integrating the CO_2_ mass flow,

$$q_{\mathrm{ads}}^{*}=\frac{F}{m_{s}}\left[{\int_{0}^{t*}\left(C_{\mathrm{in}}-C_{\mathrm{out}}\right)|}_{\mathrm{ads}}dt-{\int_{0}^{t}\left(C_{\mathrm{in}}-C_{\mathrm{out}}\right)|}_{\mathrm{bypass}}dt\right]-\frac{V_{D}C_{\mathrm{in}}}{m_{s}}$$ 1

where, *q*_ads_^*^ is the adsorption per mass of sorbent at equilibrium time t*, *C*_in_ and *C*_out_ correspond to inlet and outlet CO_2_ concentration, R is gas constant, *m*_s_ is a mass of sorbent, F is volumetric gas flow, *V*_d_ is a dead volume of the sample glass tube. Equation 1 was modified after Wilkins et al.^51^ to account for the dead volume of the sample glass tube that can be bypassed, assuming that the bypass itself has 0 volume (last term). Due to the diluted concentration of CO_2_, the inlet and outlet volumetric gas flows are assumed to be equal. In addition, the contribution of *V*_D_ to the *q*_ads_^*^ is negligible and can therefore be disregarded. The first integral (“ads”) corresponds to the adsorption stage, while the second integral (“bypass”) corresponds to the blank adsorption stage through the bypass.

During desorption, the bypass integral and *V*_D_ can be disregarded as the column is already saturated with CO_2_ of *C*_*in*_ concentration, gas lines are cleaned with pure N_2_, and the contribution of *V*_D_ is negligible. The mass balance is calculated as follows:

$$q_{\mathrm{ads}}^{*}=\frac{F}{m_{s}}{\int_{0}^{t*}\left(C_{\mathrm{in}}-C_{\mathrm{out}}\right)|}_{\mathrm{des}}\;dt$$ 2

Before desorption, dry nitrogen is flushed through the bypass to remove all of the CO_2_ in the lines before redirecting the flow through the column.

The detailed formula used to calculate *q*_ads_^*^, including the propagation of uncertainties from the individual measured variables, was analyzed to estimate the measurement errors and is presented in the Supporting Information.

### 2.3 CO_2_ Breakthrough Kinetic Modeling

Breakthrough curves were modeled with RUPTURA software^52^ using experimentally derived densities of adsorption particles, including both envelope and skeletal densities (see Supporting Information Tables S1 and S2). As the size fraction of the aerogel samples was hard to control, kinetic modeling was performed only for silica xerogels for a particle diameter of 400 μm (upper limit of the sieved fraction). Optical microscopy images of grounded xerogels are shown in the Supporting Information. It was assumed that CO_2_ adsorption isotherms follow a linear adsorption regime for silica xerogels within the range of 0–400 ppm of CO_2_; therefore, Henry isotherm was used to describe the adsorption in all materials. Breakthrough fit parameters and a detailed description of the models are presented in the Supporting Information. The respective Henry coefficient (*H*_CO2_, [mol kg^–1^ Pa^–1^]) for each experiment was obtained using the total CO_2_ uptake during a breakthrough experiment (*q*_ads_^*^) and the CO_2_ pressure at 40 Pa (*p*_400ppm_):

$$H_{\mathrm{CO}2}=\frac{q_{\mathrm{ads}}^{*}}{p_{400\mathrm{ppm}}}$$ 3

## 3 Results and Discussion

### 3.1 The Impact of Functionalization and Drying on Gel Texture

According to the IUPAC classification, all silica gel samples synthesized in this study are mesoporous.^29^ The nitrogen adsorption isotherms of the selected samples are shown in Figure 3. The isotherms are of type IV, with a hysteresis loop of type H2­(b), which is associated with pore blocking and a large distribution of neck widths.^29^ Only the isotherm measured on 2amino-16C-TMS-3Vol.-Aero could be interpreted as a hybrid type IV with type III isotherm. That sample has a hysteresis H3 loop type, which is characteristic of pore networks that include macropores that are not completely filled by the condensate.^29^ This aligns well with the goal of creating a wider porosity by adding ethanol just before gelation.

**Figure 3:**
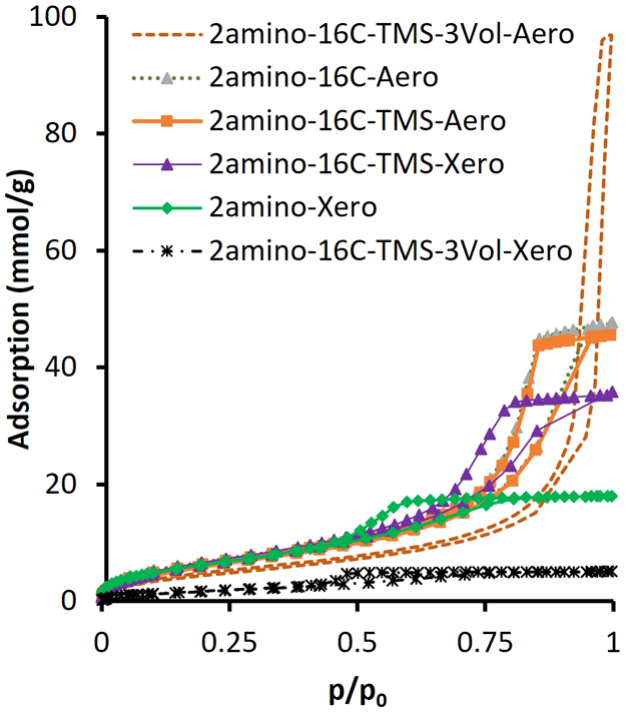
Example nitrogen adsorption isotherms measured on silica gels at 77 K.

Textural parameters (*S*_BET_, *V*_total_N2_, *d*_av_) calculated from the nitrogen adsorption isotherms are presented in Table 1. Compared to supercritical drying, ambient pressure drying systematically caused an increase in envelope density and a decrease in *S*_BET_, total pore volume, and average pore size, as expected. For the higher density samples, the partial pore collapse during ambient drying is compensated by the creation of macroporosity (wider than available from N_2_ sorption measurements; compare for skeletal and envelope density vs *V*_total_N2,_), while for initially very low-density samples (3Vol), the pore collapse leads to a much higher envelope density and decreased total pore volume. This indicates that the less dense silica backbone could not withstand the capillary forces during ambient pressure drying, almost leading to the collapse of the pore network (compare 2amino-16C-TMS-Xero and 2amino-16C-TMS-3Vol.-Xero; Table 1). On the other hand, for the samples dried in supercritical CO_2_, where capillary forces are nonexistent, the additional portion of ethanol mixed with the sol significantly increased both total pore volume and average pore size, but negatively affected the pore surface of the aerogels.

**Table 1:** Texture Parameters Measured with Nitrogen Adsorption, Chemical Composition (Carbon and Nitrogen), and Envelope and Skeletal Densities (see Supporting Information for Details)

sample name	*S*_BET_, m^2^/g	*V*_total_N2,_ cm^3^/g	*d*_av_ (4 V/*S*_BET_ by BET), nm	C (wt %)	N (wt %)	envelope density^a^ (g/cm^3^)	skeletal density^a^ (g/cm^3^)
2amino-16C-TMS-Aero	534	1.57	12	24.9	4.9	0.425	1.453
2amino-16C-Aero	551	1.65	12	19.5	3.5	0.654	1.474
2amino-16C-TMS-2Vol-Aero	379	2.18	23	27.8	4.8	NA	NA
2amino-16C-TMS-3Vol-Aero	398	2.97	30	24.8	4.5	0.215	1.455
3amino-16C-TMS-Xero	498	1.19	10	23.3	4.7	NA	NA
2amino-16C-TMS-Xero	316	0.82	10	24.9	3.5	0.513	1.432
2amino-16C-Xero	408	0.65	6	NA	NA	NA	NA
2amino-16C-TMS-3Vol-Xero	150	0.18	5	27.2	4.0	0.885	1.429
2xConc.-2amino-TMS-Xero	680	1.06	6	14.4	5.7	0.686	1.658
2amino-Xero	539	0.62	5	14.1	6.1	0.757	1.645
Lewatit VP OC 1065	NA	NA	N.A	79.2	7.6	0.540	1.126

a Description of density measurements is shown in Supporting Information.

Grafting of amine groups resulted in 4–6 wt % of elemental nitrogen for all the tested samples, based on elemental analysis (Table 1). Similar nitrogen loadings were reported previously for the silica gels synthesized using an analogous grafting route of aminopropyl groups.^36^ The higher concentration of 2amino-Si­(OMe)_3_ used during the grafting of the 2Conc.-2amino-TMS-Xero sample did not result in significantly higher nitrogen loading, possibly indicating surface saturation with amino groups. Counterintuitively, TAMS grafting did not significantly enhance the N loading despite one molecule having three amino groups instead of two. The sample functionalized with only 2amino-Si­(OMe)_3_ contained 14.1 wt % of carbon. Additional grafting of the 16C-Si­(OMe)_3_ groups (2amino-16C-Aero) increased the C content up to 19.5%. Subsequent grafting of trimethylsilyl groups increased C wt % to values around 25%, indicating some of the alkoxy or silanol groups are still present after hydrophobization with 16C-Si­(OMe)_3_. Hydrophobization with only HDMS leads to similar C wt % loading as hydrophobization with 16C-Si­(OMe)_3_ and HDMS.

Silica gel simply functionalized with 2amino-Si­(OMe)_3_, without any hydrophobization, retained a significant fraction of the initial porosity even after drying under ambient conditions (compare 2Conc.-2amino-TMS-Xero and 2amino-Xero; Table 1). This is remarkable as hydrophobization is usually required to maintain high porosity.^53^ Most likely, the high silica concentrations help the network to resist shrinkage.^54^ Indeed, the 2amino-16C-TMS-3Vol-Xero sample, produced from a more diluted sol, has a higher density, despite hydrophobization treatment. In addition to the effects of sol concentration, the propylethylenediamine chains seem to have an effect similar to hydrophobization in preventing silica condensation at particle–particle interfaces during drying, which in turn allows a partial “spring-back” of the silica-particle structure at the end of the drying.^32^

The ^1^H–^29^Si cross-polarization MAS NMR spectra are shown in Figure 4A. The bands at −109 and −103 ppm correspond to silica coordinated by with 4 bridging oxygen atoms (Q^4^) and 3 bridging oxygen and 1 nonbridging oxygen atoms (Q^3^), respectively. These oxygen bridges link the central Si atom to 4 (Q^4^) or 3 (Q^3^) other silica tetrahedra. In the case of Q^3^, the silicon tetrahedron is also coordinated by another nonbridging oxygen (silanol or alkoxy groups).^49^ The cross-polarization disproportionally enhances the intensity of the Q^3^ signal over that of Q^4^ and the peak areas are thus not quantitatively related to the actual Q^3^ and Q^4^ concentrations. Nevertheless, the relatively high surface area of Q^3^ with respect to the Q^4^ band for 2amino-Xero suggests a relative abundance of hydrophilic silanol or water-reactive ethoxy groups. The Q^3^ band is significantly less pronounced than the Q^4^ band for all of the other samples. The band at −68 ppm corresponds to a silicon atom in the tetrahedron coordinated by the C atom of the functional group and 3 bridging oxygen atoms (T^3^). This confirms the successful grafting of 2amino- groups in the case of 2amino-Xero and 2xConc.2amino-TMS-Xero samples. The surface of the same band for 2amino-16C-TMS-Aero and–Xero samples is ca. 2 times higher, which also suggests successful grafting of aliphatic groups in addition to 2amino- groups. The shoulder of the −68 ppm band (∼−60 ppm, T^2^) probably corresponds to the same chemical environment as for the −68 ppm band, except the silicon tetrahedron is coordinated by 2 bridging oxygen atoms and 1 non bridging oxygen. The band at 12 ppm is related to the TMS group. It confirms the successful grafting of that group for the 2xConc.-2amino-TMS-Xero, 2amino-16C-TMS-Xero, and 2amino-16C-TMS-Aero samples;^49^ however the grafting efficiency of TMS groups on the 2amino-16C-TMS-Aero was significantly lower comparing to the corresponding 2amino-16C-TMS-Xero sample.

**Figure 4:**
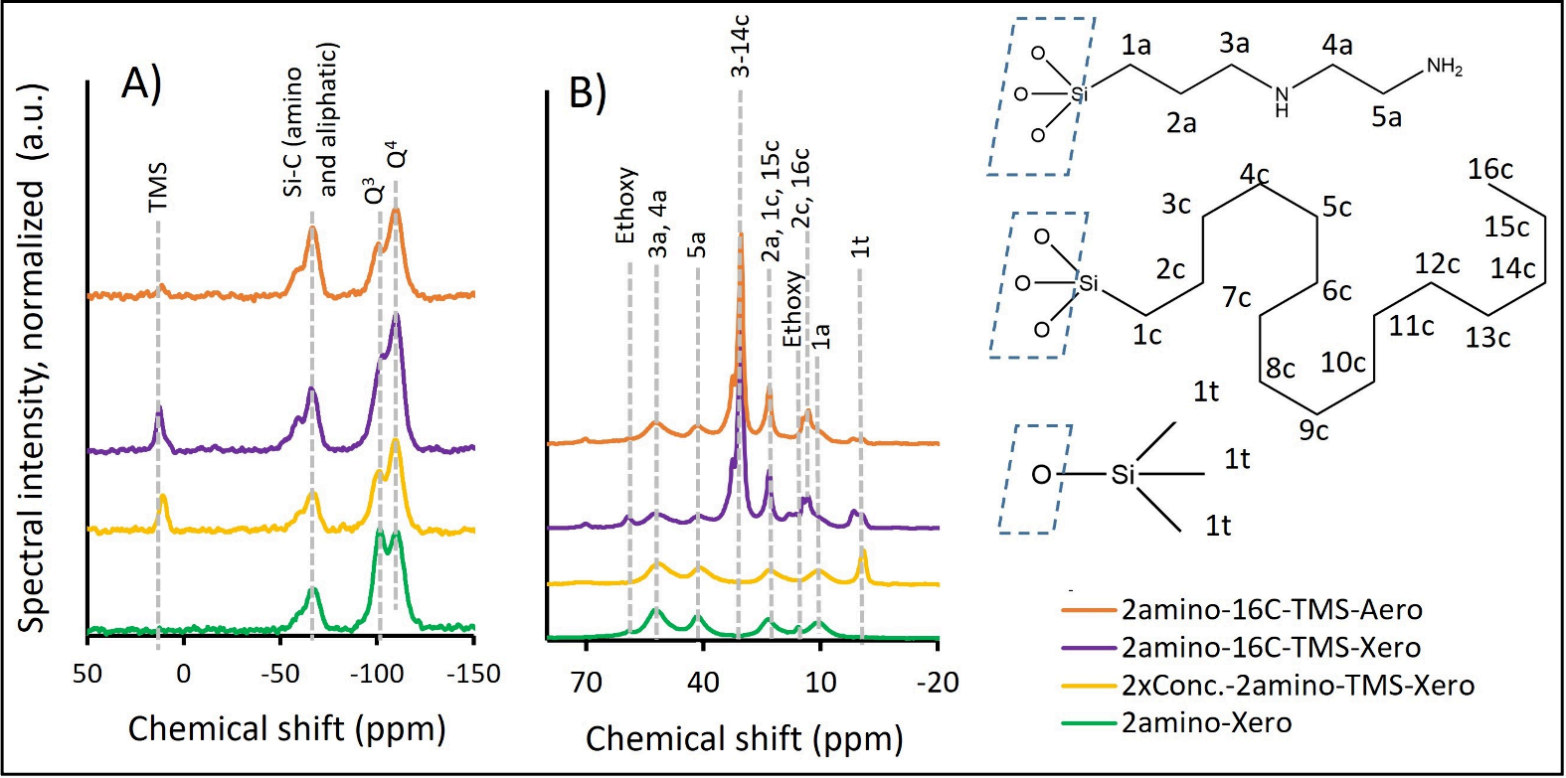
Solid-state NMR spectra: (A) nonquantitative one-dimensional ^1^H–^29^Si cross-polarization; (B) quantitative ^13^C spectra. Spectra are normalized to the same number of scans and same mass of the sample in the rotor.

The quantitative, single-pulse ^13^C NMR spectra are shown in Figure 4B. The ^13^C spectra predictions used for band assignment were made using ChemDraw 23.0 software (Revvity Signal Software, Inc.). The predicted spectra are shown elsewhere (Supporting Information Figures S2 and S3). For the 2amino-Xero sample, all four bands for carbon atoms in the 2amino- group are visible at: 50 ppm (3a and 4a; Figure 4B), 40 ppm (5a; Figure 4B), 24 ppm (2a; Figure 4B), and 10 ppm (1a; Figure 4B). The band at −1 ppm corresponds to the TMS group and confirms the TMS grafting for all the samples hydrophobized with HDMS. For 2amino-16C-TMS-Xero and–Aero samples, however, this band has a significant shoulder, indicating that for the samples hydrophobized with 16C-Si­(OMe)_3_ and HDMS, the chemical environment of TMS groups is more complex than for the sample hydrophobized only with HDMS. For 2amino-16C-TMS-Aero and–Xero samples, a distinctive band is visible at 30 ppm, which corresponds to c3–14 carbons in the aliphatic chain of 16C-Si­(OMe)_3_, confirming successful grafting of that aliphatic chain to the surface.

The broad nature, irregular shape, and overlap of all bands significantly hinder the precise quantification of grafted groups, not allowing for precise deconvolution and quantification of individual groups. The total carbon and nitrogen content calculated based on the integration of the 5a band (Figure 4B), together with grafting densities of 2amino, TMS, and 16C groups, are shown in Table 2. *S*_BET_ was used for grafting densities calculations. The 5a band was chosen for calculations of the total nitrogen concentration and grafting density of 2amino- groups because of the least significant peak overlap. For calculations of the grafting densities of the 16C group, a band at 30 ppm was used.

**Table 2:** Total Carbon and Nitrogen Loading Estimated from the ^13^C NMR Method with Grafting Densities of Propylethylenediamine, Trimethylsilyl, and Hexadecyl^a^

sample name	total C (wt %), ^13^C NMR	C (wt %), elemental	N (wt %),^a^ ^13^C NMR	N (wt %), elemental	grafting density 2amino, mmol/g [groups/nm^2^]^a^	grafting density TMS, mmol/g [groups/nm^2^]	grafting density 16C, mmol/g [groups/nm^2^]
2amino-16C-TMS-Aero	22.1	24.9	4.1	4.9	1.5 [1.6]	0.1 [0.1]	0.8 [0.8]
2amino-16C-TMS-Xero	22.8	24.9	2.9	3.5	1.0 [1.9]	0.3 [0.6]	0.8 [1.5]
2xConc.-2amino-TMS-Xero	10.7	14.4	5.3	5.7	1.9 [1.7]	0.4 [0.4]	
2amino-Xero	11.2	14.1	5.6	6.1	2.0 [2.2]		

a Total carbon and N loadings calculated using the NMR method were compared to C and N concentrations from elemental analysis (see Table 1).

b Calculated based on 5a carbon peak. Note that there are significant uncertainties with these data due to the peak overlap.

Compared to the elemental analysis results, values estimated from NMR (total C wt % and N wt %) are systematically lower (by 2.1–3.7% for carbon, 0.4–0.8% for nitrogen). The most significant differences are for nitrogen content in 2amino-16C-TMS-Aero and -Xero samples, for which the 5a band is significantly overlapping with the 3–14c band (Figure 4B). The total grafting densities of 2amino groups are similar between samples (1.9–2.2 groups/nm^2^_;_ Table 2). The following treatment with HDMS resulted in grafting of 0.4 groups/nm^2^ for 2xConc.-2amino-TMS-Xero sample, and 0.1 and 0.6 groups/nm^2^ for 2amino-16C-TMS-Aero and -Xero samples, respectively. The hydrophobization with 16C aliphatic chains led to grafting of 0.8 and 1.5 chain/nm^2^ for 2amino-16C-TMS-Aero and -Xero samples, respectively. However, as the change in the *S*_BET_ for the 2amino-16C-TMS-Xero sample could be related to a pore network shrinkage after drying, therefore a potential decrease in N_2_ accessibility at cryogenic temperature, a portion of the 2amino, TMS, and 16C densities (per nm^2^) could be overestimated, because initially during the functionalization and hydrophobization, 2amino-16C-TMS-Aero and–Xero samples should have similar accessible surface area.

### 3.2 Water Sorption, Texture, and Surface Chemistry

High water adsorption, resulting from the inherent water affinity of the hydrophilic silanol groups terminating the silica backbone, is a major challenge for the use of silica gels as DAC sorbents. Moreover, amino groups grafted on silica surfaces are also highly hydrophilic. Poor CO_2_/H_2_O selectivity and high regeneration costs can result from resaturating the pores with water. Resaturation may also cause pore blocking and compromise the fragile nanostructure of unmodified hydrophilic aerogels, which cannot endure significant capillary forces.^55,56^ We speculate that the scarcity of the available CO_2_ and H_2_O cosorption data under high RH on potential DAC sorbents, including silica gels, stems from these limitations.

When exposed to high RH expected for DAC (>80%), it is anticipated that mesopores of silica gels will be filled with water due to capillary condensation. To estimate the pore diameter filled with water, one can use the Kelvin–Cohan equation:^57^

$$r-t=-\frac{2\gamma \cos\theta V_{m}}{RT\ln\left(\mathrm{RH}\right)}$$ 4

where *r* is the radius of the pore, γ is the surface tension of water (72.0 mJ/m^2^ at 25 °C), *V*_m_ is the molar volume of liquid water (18.07 × 10^–6^ m^3^/mol), θ is a contact angle, and *t* is statistical adsorbed layer thickness (0.2–0.4 nm at 80% RH and 0.4–0.8 nm at 90% RH for silica).^58^

Silica gel samples that are not hydrophobized have a contact angle similar to those functionalized with aminopropyl groups, which is close to zero.^31^ Thus, for these samples, perfect wetting can be assumed. Pores with 10 and 20 nm diameters should be filled with water under 80, and 90% RH, respectively. Indeed, as presented in Figure 5A, the 2amino-Xero sample reached ∼50 wt % of water sorption at 90% RH (∼0.5 cm^3^ of liquid water/g of sample), with significant capillary condensation occurring above 70% RH. The maximum sorption recorded for this sample is close to the total porosity of the sample measured with nitrogen adsorption (0.6 cm^3^/g; ca. 0.5–100 nm; Table 1), indicating a close to complete filling of the pore network by water. Almost complete water saturation of the nonhydrophobized pore network with a characteristic pore size of 5 nm is in agreement with the calculations using eq 4.

**Figure 5:**
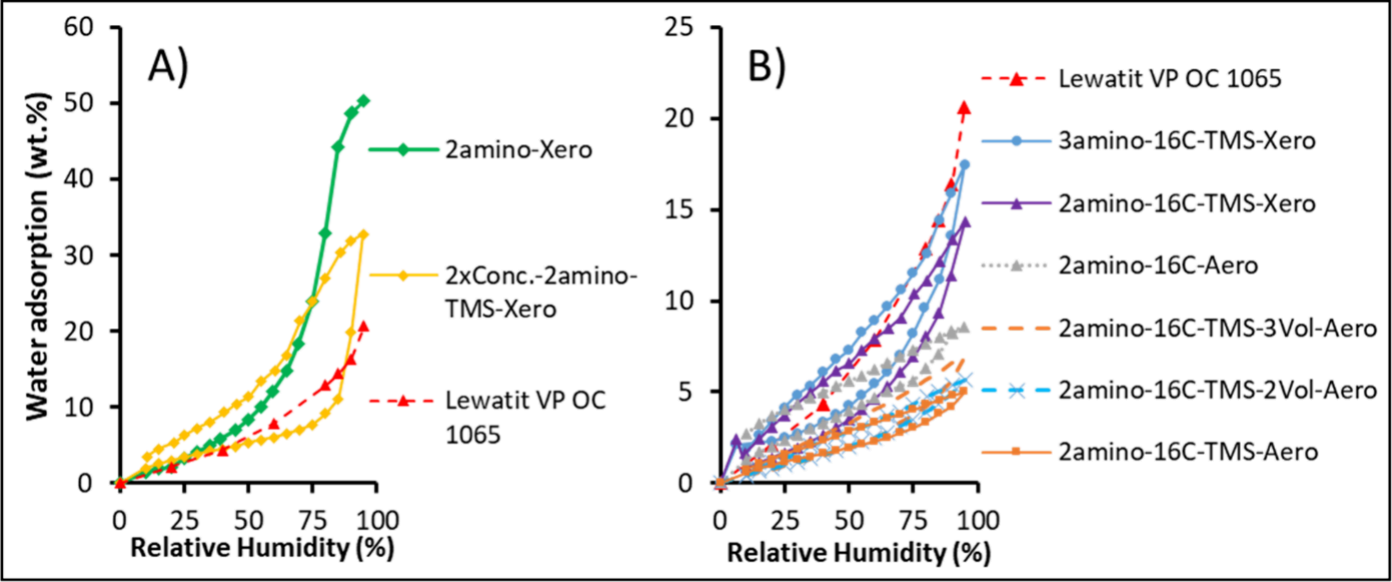
Water adsorption isotherms measured on silica gels and a Lewatit VP OC 1065. For Lewatit and 2-amino-Xero, only the adsorption branch was measured. For clarity, samples are separated between high mass uptake (A) and low-to-moderate mass uptake (B).

Silica gel hydrophobized with HDMS only (in addition to the amino functionalization present in all samples) displayed a relatively low water uptake up to 70% of RH (∼7 wt %), but water capillary condensation occurred for that sample at 80% RH (Figure 5A). By comparison of *V*_total_N2_ and H_2_O sorption for that sample, only 30% of the pore network volume was filled. This is even though the pore size distribution of 2Conc.-2amino-TMS-Xero is similar to that of 2amino-Xero, with an average pore diameter of 6 nm (Figure 6, Table 1). Indeed, it has been shown that grafting of the trimethylsilyl groups results in hydrophobic surfaces with a water contact angle of 128°.^31^ However, as capillary condensation still occurs, the pore surface of 2Conc.-2amino-TMS-Xero is not completely hydrophobic due to the presence of hydrophilic diamino groups.

**Figure 6:**
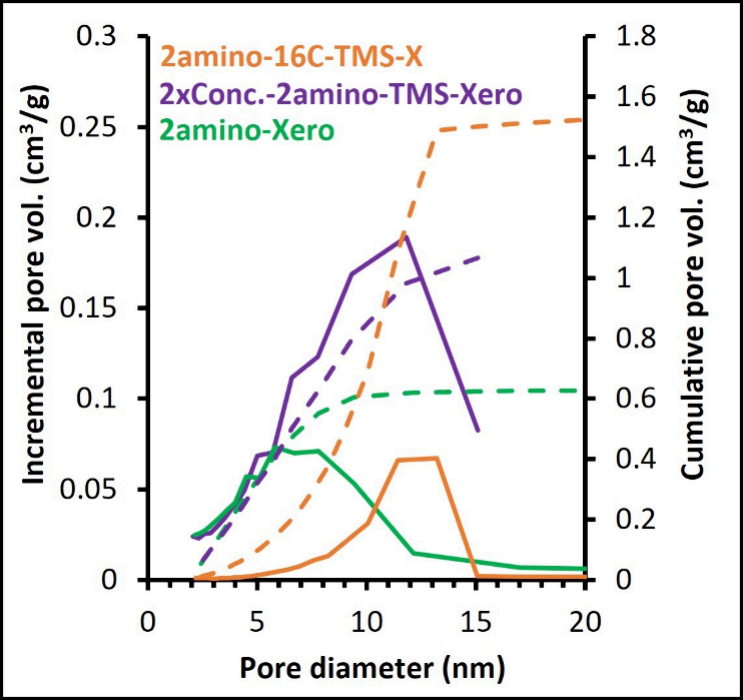
Examples of the pore size distribution calculated using the BJH method. Solid and dashed lines represent incremental and cumulative pore volume, respectively.

The silica aerogels with double hydrophobization with hexadecyl and trimethylsilyl presented the lowest H_2_O uptakes, with a maximum of ∼6 wt % under 95% RH. In the tested RH range, the adsorption isotherms were close to linear with minimal hysteresis and the H_2_O maximum sorption was very low relative to the pore volume (Table 1), indicating that capillary condensation did not occur, and the pore surfaces remained hydrophobic. This is further supported by the water contact angle of 161 °C measured on the 2amino-16C-TMS-3Vol-Aero sample, classifying it as a superhydrophobic material (Supporting Information, Figure S10). The water sorption on the silica aerogels with double hydrophobization is likely associated with the hydrophilic active sites (amines) that are still present on the surface. Nonetheless, long aliphatic chains compensate for the hydrophilic nature of the amine sites, potentially screening them geometrically, which prevents bulk capillary condensation. Similar H_2_O uptake was recorded for xerogel 2amino-16C-TMS-3Vol.-Xero having different isotherm shape. Here, due to pore volume contraction (0.18 cm^3^/g, Table 1), the adsorbed volume of water filled ∼40% of the total pore volume measured with N_2_ sorption.

The H_2_O uptakes on the other silica xerogels with double hydrophobization were comparatively low for RH below 50% (up to ∼3 wt %, Figure 5B). At RH above 50%, the uptakes rose more steeply, with a 3 times higher maximum uptake at 95% RH with respect to the 16C-TMS-Aero samples. The change of adsorption curvature above 50% RH and the occurrence of hysteresis for these samples indicate that capillary condensation only fills a fraction of the narrowest pores (e.g., ∼17% pore volume filled with water at 95% RH of 2amino-16C-TMS-Xero; Table 1, Figure 5B).

The silica gel sample hydrophobized only with 16C-Si­(OMe)_3_ showed only slightly higher water uptakes than the corresponding sample with double hydrophobization (compare 2amino-16C-Aero and 2amino-16C-TMS-Aero; Figure 5), as well as a significant kinetic-limited hysteresis at <40% RH (based on transient data profile) that was not observed in samples with double hydrophobization.

Aerogels and xerogels with double hydrophobization showed significantly lower water uptake than Lewatit VP OC 1065 in the whole RH range (Figure 5). The sample hydrophobized with HDMS displayed comparable water uptakes to Lewatit up to 90% RH, but significantly exceeded it at 95% RH. The nonhydrophobized sample showed water uptake similar to Lewatit up to 50% RH but much higher than for Lewatit beyond that RH.

### 3.3 Effects of Water Adsorption on CO_2_ Adsorption

Figure 7 presents the single-component CO_2_ adsorption isotherms obtained at 25 °C using volumetric dosing. All the measured CO_2_ isotherms are of type I.^29^ The interpolated sorption values at 0.4 mbar, corresponding to 400 ppm of CO_2_ at 1 bar, are given alongside the sample names. All the samples of silica gels, except the 2amino-16C-TMS-3Vol.-Xero, showed similar sorption values, between 0.2 and 0.3 mmol/g at 0.4 mbar, which should correspond to 20–40% of the amino groups being involved in the formation of the carbamate (2R_2_NH + CO_2_ → R_2_NCO_2_^–^:R_2_NH_2_^+^) thought to be at the origin of the CO_2_ adsorption,^59^ considering the typical grafting densities (Table 2). For 2amino-16C-TMS-3Vol.-Xero, though, low CO_2_ sorption was measured at low pressures. In contrast, values similar to those of the other silica gels were reached at higher pressures (>10 mbar). However, the chemical composition of this sample is very similar to that of the corresponding 2amino-16C-TMS-Xero sample. As discussed above, the former suffered a partial pore collapse during drying (Table 1). Therefore, kinetic limitations leading to nonequilibrium measurement conditions likely explain the difference at low pressure. The sorption on Lewatit VP OC 1065 under 0.4 mbar was 2–3 times higher than that on functionalized silica gels.

**Figure 7:**
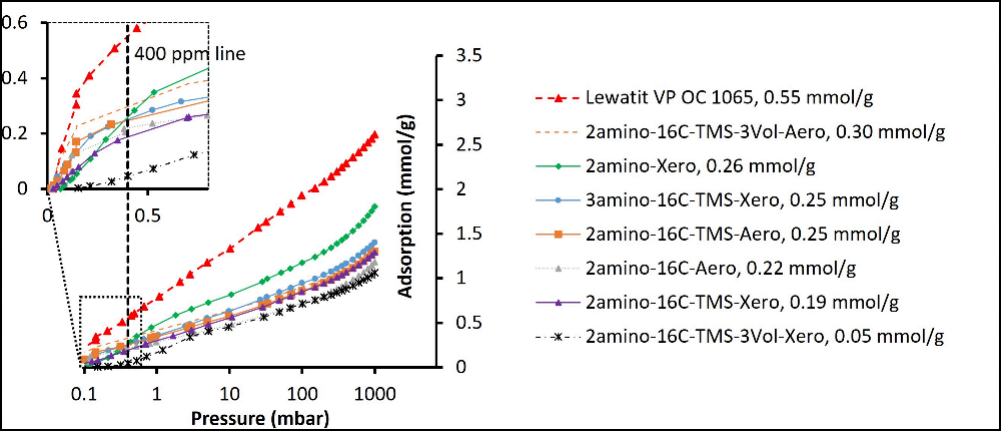
Single component CO_2_ adsorption isotherms measured on silica gels at 25 °C.

The calculated uptakes of CO_2_ in breakthrough experiments for each RH step are presented in Figure 8. The cumulative CO_2_ sorption was calculated for each relative humidity step in first breakthrough cycle using eq 1 (the 1 cycle measurement is shown in Figure 2A). For Lewatit VP OC 1065, the CO_2_ adsorption measured under dry conditions matches the value obtained by the volumetric method (0.55 mmol/g, volumetric; Figure 7, cf. 0.547 mmol/g, breakthrough; Figure 8). In contrast, the CO_2_ adsorption measured on silica gels with double (16C-Si­(OMe)_3_ and HDMS) hydrophobization was systematically higher when calculated from the breakthrough experiments at 0% RH than the values obtained by volumetric experiments (∼0.32 mmol/g; Figure 8, cf. 0.2–0.3 mmol/g in the volumetric experiment; Figure 7). A possible explanation of the discrepancy is that the CO_2_ adsorption values measured on silica gels in volumetric experiments were not entirely in equilibrium because of slower uptake kinetics compared to Lewatit, which can be due to both particle size and diffusivity within the sample. Specifically, samples subjected to the volumetric method, in contrast to breakthrough experiments, were not ground, and the particle size of gels (8 mm) was much higher than for Lewatit (∼500 μm).

**Figure 8:**
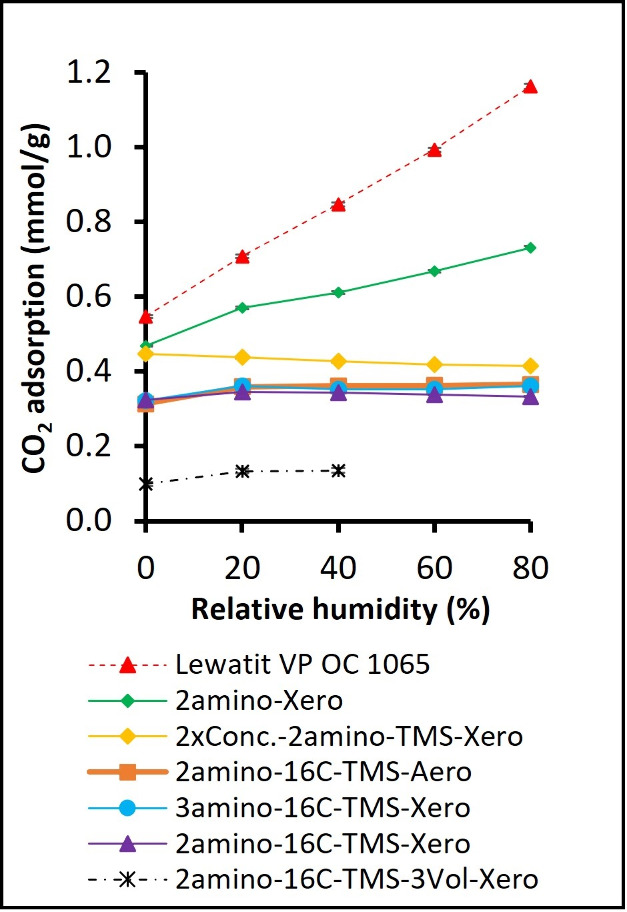
CO_2_ adsorption was measured during the breakthrough experiments (400 ppm of CO_2_) under varying relative humidity. The CO_2_ sorption was evaluated for each relative humidity step in the 1st breakthrough cycle, Figure 2A.

The nonhydrophobized sample, as well as the sample hydrophobized only with HDMS, showed a ∼40% higher CO_2_ adsorption under dry conditions compared to samples with double hydrophobization (Figure 8). For only two samples, increasing RH significantly increased CO_2_ adsorption: in the benchmark Lewatit VP OC 1065 resin and nonhydrophobized 2amino-Xero. The sample hydrophobized with HDMS only (2xConc.-2amino-TMS-Xero) shows a relatively constant CO_2_ adsorption, slightly decreasing with increasing RH.

All other silica gel samples with cografted aliphatic and trimethylsilyl groups (16C-Si­(OMe)_3_ and HDMS) show a relatively minor increase in CO_2_ adsorption when RH is ramped from 0 to 20%. For RH above 20%, the measured uptakes are constant and generally overlap within the error bars in the whole range, except for the outlier 2amino-16C-TMS-3Vol.-Xero, with 3 times lower CO_2_ uptake, likely as a result of the collapsed pore network.

Silica gels, unlike Lewatit, do not exhibit swelling upon sorption, which can be advantageous for the shaping and structural stability of sorbent materials. During breakthrough experiments, it was observed that Lewatit undergoes significant swelling within the capillary upon exposure to elevated relative humidity, although this effect was not quantified. Such swelling behavior can present notable disadvantages for contactor design, including an increased pressure drop, reduced mechanical stability, and potential deformation of the sorbent bed. In contrast, the dimensional stability of silica under varying humidity conditions makes it a more predictable and robust candidate for engineered contactors.

The amounts of adsorbed and desorbed CO_2_ in the first (RH subsequently ramped from 0 to 80%; Figure 2A) and second cycle (after saturation with 80% RH; Figure 2B) are shown in Figure 9. For all of the samples, we observe that the CO_2_ sorbed during the adsorption step of the first cycle was entirely released during desorption. The values of CO_2_ sorbed and desorbed after saturation with water were similar to those from the first cycle, except for the silica gel without any hydrophobization, where water condensation blocked the active sites for CO_2_ sorption. Thus, while double hydrophobization slightly decreases the level of CO_2_ uptake under dry conditions (Figure 8), it significantly improves the material’s ability to retain sorption capacity under humid conditions after saturation with 80% RH (Figure 9).

**Figure 9:**
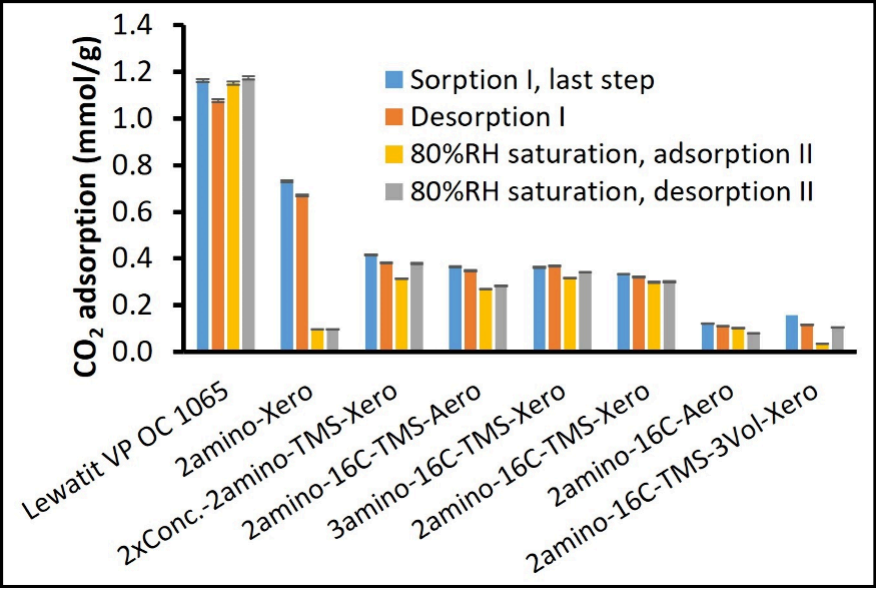
Comparison of adsorbed (summarized for all relative humidity steps) and total desorbed CO_2_ amount in the first and second cycles of the breakthrough (400 ppm of CO_2_). Note that the 2nd cycle was carried out after saturation with water (80% relative humidity, without CO_2_).

### 3.4 Selectivity of Silica Sorbents under DAC Conditions

The CO_2_/H_2_O sorption selectivity of silica gels and Lewatit VP OC 1065 in mol of CO_2_/ mol of H_2_O adsorbed is presented in Figure 10. It was calculated based on eq 5:

$$S_{\mathrm{CO}2/H2O}=\frac{q_{\mathrm{ads}}^{*}}{w_{\mathrm{ads\_}H2O}^{*}}$$ 5

where *S*_CO2/H2O_ is CO_2_/H_2_O sorption selectivity, q*_ads_ is CO_2_ adsorption measured for each relative humidity step in the breakthrough experiments in first sorption cycle, as presented in Figure 2A, and w*_ads_H2O_ is water adsorption for each relative humidity step. Due to the high uncertainty of the computed water adsorption in the breakthrough setup, particularly for hydrophobic samples, the water sorption was separately quantified in single-component H_2_O sorption experiments. It was shown, however, that CO_2_ sorption does not affect the H_2_O sorption on the amine-modified samples, including Lewatit VP OC 1065.^60,61^ In Figure 10, yellow and blue indicate the usual RH values in Europe in summer and winter, respectively.^15^ The CO_2_/H_2_O selectivity presented in Figure 10 was calculated for relatively unfavorable conditions characterized by high relative humidity and a relatively high average temperature. Notice that the average temperature in Europe is not 25 °C and CO_2_ sorption at lower temperatures is expected to be significantly higher (please refer to Supporting Information, Figure S1), and because of the higher heat of CO_2_ adsorption on amine-functionalized materials with respect to heat of H_2_O adsorption.^13,14^ Higher heat of adsorption means that the equilibrium pressure is more sensitive to temperature increases, resulting in a more pronounced drop in adsorption when the temperature rises.^62^

**Figure 10:**
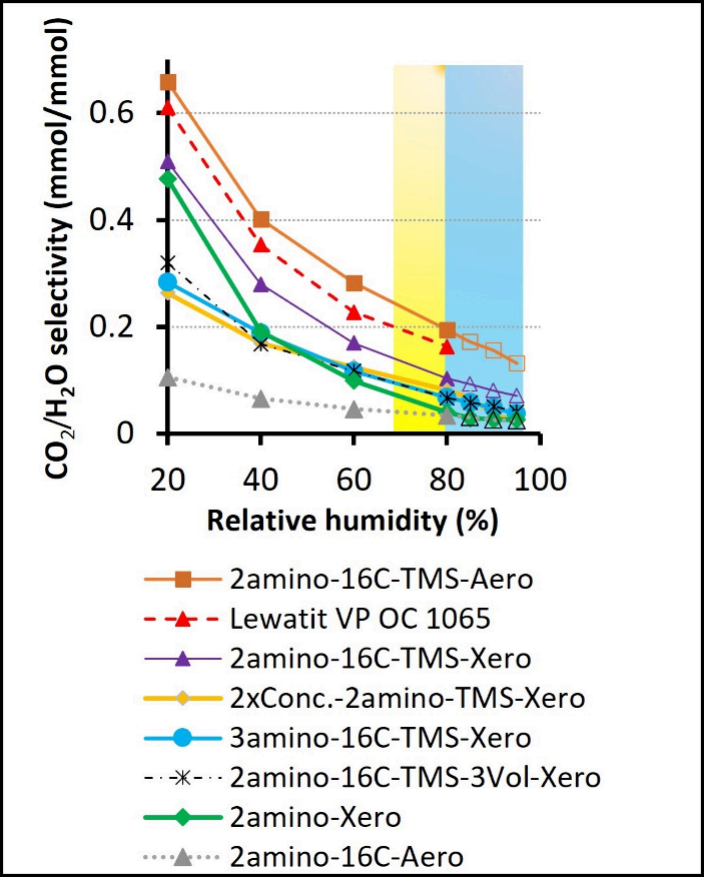
CO_2_/H_2_O adsorption selectivity on silica gels and Lewatit VP OC 1065 (400 ppm of CO_2_). The data are calculated based on the CO_2_ adsorption measured in the breakthrough experiments for each individual RH step in the first cycle. Water adsorption was quantified during the separate dynamic vapor sorption experiments. Yellow and blue colors indicate usual RH values in Europe in summer and winter, respectively (New et al. 2002 ^15^). Data points above 80% of RH for silica gels were extrapolated, assuming constant CO_2_ uptake measured at 80%, utilizing relatively constant CO_2_ sorption as a function of RH (the symbols without filling; cf. Figure 8).

The Lewatit VP OC 1065 showed high selectivity toward CO_2_, ranging from 0.60 mol CO_2_/mol H_2_O under 20% RH to 0.16 under 80% RH. In the same range, the most selective silica gel synthesized in this study (2amino-16C-TMS-Aero) showed selectivity ranging from 0.66 to 0.20 mol CO_2_/mol H_2_O, corresponding to an increase between 10 and 25% with respect to the Lewatit sample. The selectivity of xerogel with double hydrophobization, 2amino-16C-TMS-Xero was lower than the Lewatit sample by 25% at 60% RH and 35% at 80% RH, respectively. This selectivity might still be considered acceptable because the ambient drying method allows for production costs much lower than those of supercritical drying. The CO_2_/H_2_O selectivity of other silica gels at RH > 80%, especially the nonhydrophobized 2amino-Xero gel, is significantly lower, clearly questioning their use for DAC.

The nonhydrophobized 2amino-Xero showed one of the lowest selectivities at 80% RH. Given that the CO_2_ sorption values for this sample after water saturation (80% RH) were ∼7 times lower than in the first breakthrough cycle, where RH was incrementally increased (Figure 9), even the values presented in Figure 10 for this sample are likely overestimated. This suggests that CO_2_ was unable to compete with the large amount of water adsorbed due to capillary condensation (see Figure 5A) or that CO_2_ diffusion was significantly hindered by water-blocked pores. To our knowledge, none of the promising silica gel sorbents for DAC application reported in the literature was hydrophobized in addition. In fact, neither the sorption of 400 ppm of CO_2_ at RH conditions near the water saturation was evaluated nor were H_2_O sorption effects accounted for under these conditions.^14^ The CO_2_ sorption behavior on 2amino-Xero emphasizes the importance of water sorption when evaluation silica gels for DAC applications. Indeed, while showing good adsorption capacity in the absence of water, hydrophilic silica gels are likely bad candidates under realistic DAC conditions.

At realistic DAC operating conditions, it is expected that the humidity is often 80% or higher during the adsorption phase. A much larger partial pressure of water with respect to CO_2_ in the ambient air will cause water saturation of the material before it can be saturated with CO_2_. Additionally, sorbent saturation with water prior to CO_2_ sorption might occur because significant quantities of water vapor are often introduced just before or during the desorption cycle to (1) increase CO_2_ cycling mass by decreasing the partial pressure of CO_2_ in the adsorption contactor and minimizing the residual CO_2_ present in the dead volume voids,^12^ (2) replace oxygen to avoid oxidative degradation of amines at temperatures above 100 °C,^63^ and (3) prevent urea formation, which deactivate the amines.^64^

### 3.5 Water Saturation and CO_2_ Sorption Kinetics

Due to the low CO_2_ concentration in the atmosphere, at least ∼10^3^ m^3^ of air must be processed through the adsorption column to capture 1 kg of CO_2_.^14^ As the pressure drop across the column is inversely proportional to the sorbent particle size,^65^ fast sorption kinetic, even under humid conditions, is highly desirable as it allows the use of larger sorbent particles and a reduction of the pressure drop. Also, fast sorbent loading and unloading cycles are crucial for reducing costs and building lightweight collectors.

Owing to the differences in the BTA experiments, namely, the length and porosity of the packed beds used, the kinetics observed cannot be compared directly. Dimensionless time derived from bed length, bed porosity, and gas velocity was used to compare these results with equalized effects from porosity and bed length (Supporting Information, Figure S4). The uptake kinetics within the silica gels was modeled to gain additional insights. The breakthrough curves were modeled by varying the linear driving force kinetic constant (*k*, Table 3, see Supporting Information, Breakthrough Modeling):

$$\frac{\partial q_{\mathrm{ads}}^{\mathrm{bead}}}{\partial t}=k\left(q_{\mathrm{ads}}^{*}-q_{\mathrm{ads}}^{\mathrm{bead}}\right)$$ 6

where *q* ^bead^_ads_ is the average CO_2_ uptake inside the bead, q*_ads_ is the equilibrium CO_2_ uptake estimated from BTA using the Henry isotherm. The simple Henry isotherm was used to avoid overfitting. For CO_2_ in the 0–0.4 mbar range (0–400 ppm), a Henry isotherm approximates adsorption well enough for kinetic modeling. However, Langmuir-type isotherm nonlinearity leads to adsorption–desorption asymmetry, particularly a broader desorption front, especially above 40 Pa, making the linear model inadequate for capturing desorption kinetics and regeneration.^66^

**Table 3:** Breakthrough Simulation Results for Dry and Humid Flows and Henry Coefficients Used

sample/state	kinetic constant *k*, s^–1^	Henry coefficient for CO_2_, *H*_CO2_, mol kg^–1^ Pa^–1^	root mean square error (0–380 ppm range) RMSE, ppm
2amino-Xero/dry	0.10	0.0118	0.4
2amino-Xero/water-saturated	NA	0.0017	0.6
2amino-16C-TMS-Xero/dry	0.01	0.0084	0.3
2amino-16C-TMS-Xero/water-saturated	0.01	0.0078	0.2
2xConc.-2amino-TMS-Xero/dry	0.01	0.0118	0.2
2xConc.-2amino-TMS-Xero/water-saturated	0.01	0.0105	0.3

Henry isotherm coefficients are presented in Table 3 and correspond to the uptakes shown in Figure 8. The optimal fit of the breakthrough curves was achieved via optimization of the root-mean-square error (RMSE):

$$\mathrm{RMSE}=\sqrt{\frac{\overset{N}{\underset{0}{\sum }}{\left(y_{i}-y\right)}^{2}}{N^{2}}}$$ 7

where *N* is the number of experimental breakthrough data points, *y*_*i*_ and *y* are the experimental and fitted CO_2_ concentrations, respectively. The final values of the root-mean-square error are also shown in Table 3. We would like to point out that the results are semiquantitative at best due to simplifications within the model, namely the assumption of spherical beads of uniform size (please refer to optical images of gels in Supporting Information) and the use of the Henry isotherm. Still, they allow us to estimate the effect of water adsorption by comparing the estimated kinetic properties at 80% RH with the kinetics under dry conditions of CO_2_. The results of the breakthrough simulations are listed in Figure 11.

**Figure 11:**
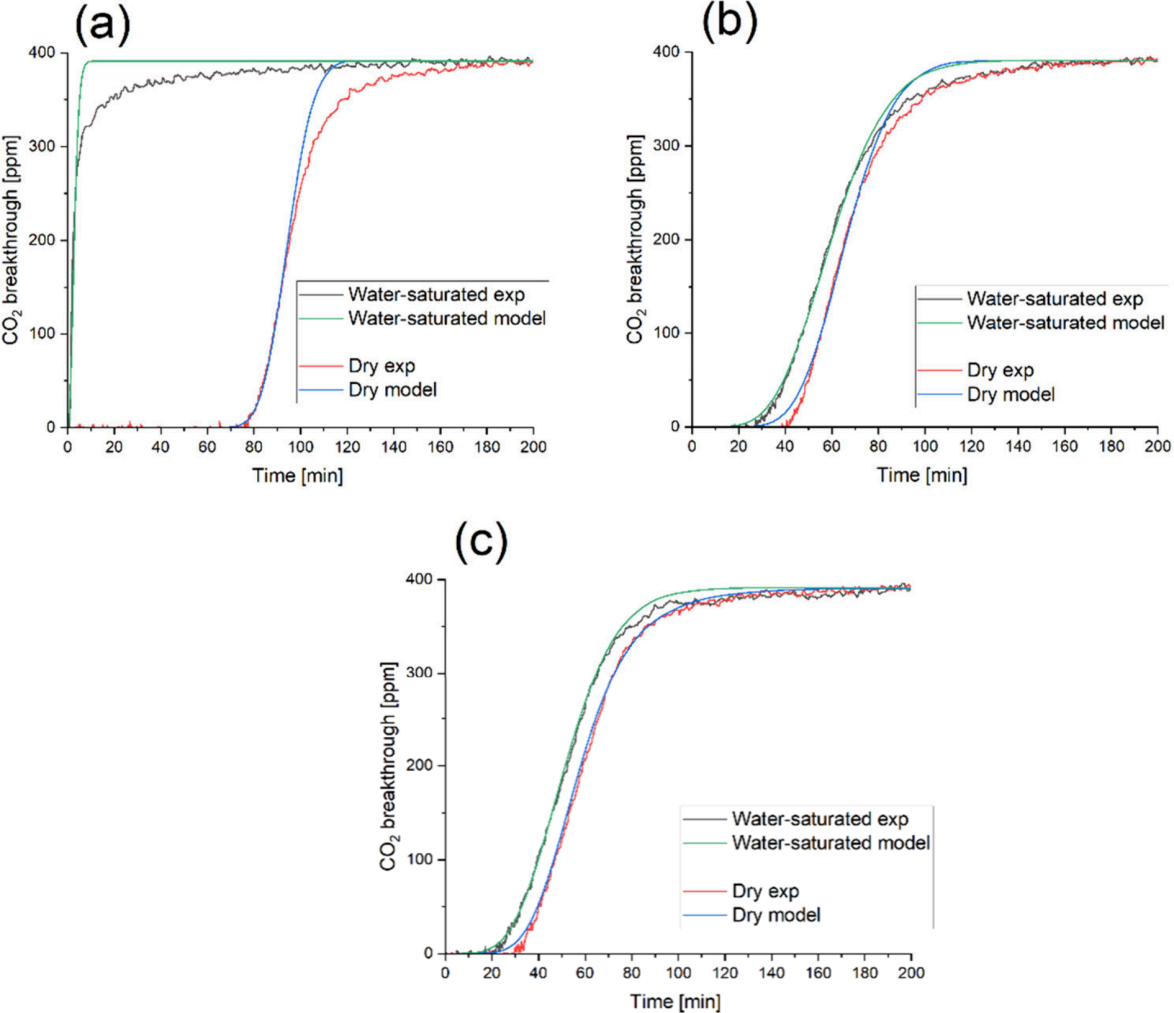
Experimental and simulated CO_2_ breakthrough curves under dry conditions and after sample saturation with 80% relative humidity prior to CO_2_ breakthrough for (a) 2amino-Xero, (b) 2amino-16C-TMS-Xero, and (c) 2xConc.-2amino-TMS-Xero.

It can be seen that the breakthrough curves are better fitted at the beginning of the breakthrough curve than at the end with CO_2_ concentrations approaching 400 ppm. A possible explanation is the particle size distribution, which has not been taken into account, where even a small number of larger particles with a correspondingly large uptake due to their large volume have a large impact. In contrast, smaller particles are less critical due to their small volume. Other factors, such as the irregular shape of the particles or slowing down of the diffusion due to pores filled with water, are likely to contribute to the deviation as well. However, it is interesting to see that the deviation between the model and measured breakthrough curve is highest for the 2amino-Xero sample and lowest for the 2xConc.-2amino-TMS-Xero. Additionally, the modeling of the 2amino-Xero is complicated by the very small uptake capacity of the sample under humid conditions and the almost instantaneous breakthrough, which leads to relatively high uncertainties.

As presented in Figure 11 and Table 3, contrary to nonhydrophobized samples, hydrophobization with HDMS allowed the slope of the breakthrough curve (thus also the kinetic constant) to be the same for silica xerogels even after exposure to 80% of RH. Hydrophobization seems to eliminate the effect of water on the uptake kinetics. For the 2amino-Xero sample, however, the model fits the experiment well under dry conditions, but it performs poorly under wet conditions due to the effects of diffusion through the water-saturated porous network. This could be attributed to pore clogging by water, which leads to an extended mass transfer zone or a long tail in the breakthrough curve (Figure 11A). Due to high modeling error, we do not report *k* for the water-saturated 2amino-Xero sample, as the calculations are unreliable.

2xConc.-2amino-TMS-Xero and 2amino-16C-TMS-Xero samples showed the same linear driving force kinetic constants k under dry and water-saturated conditions, indicating the positive effect of hydrophobization on the CO_2_/H_2_O selectivity and CO_2_ adsorption kinetics under water-rich conditions. The kinetic constants calculated for the samples near water saturation are more representative of realistic DAC conditions than those typically reported in the literature and measured under dry conditions.

The kinetic constant *k* in hydrophobized samples under dry conditions was 1 order of magnitude lower than for dry 2amino-Xero despite the similar pore size distribution, i.e., (*d*_av_ = 6 and 5 nm in 2xConc.-2amino-TMS-Xero and 2amino-Xero, respectively; see Figure 6), the same particle size distribution (400 μm), and very similar bead porosity (Table S2). This suggests that the trimethylsilyl groups affect the CO_2_ mobility through the pore network. Amino groups are known to enhance CO_2_ diffusion through silica-based microporous membranes by increasing the surface mobility of CO_2_.^67^ Since bulky trimethylsilyl groups are likely grafted in between amino groups, they might impede their mobility by limiting CO_2_ diffusion through the pore throats. However, for the 2amino-Xero sample, the slow tail of the breakthrough curve, which has not been taken into account, is much more important and could influence the conclusions of the overall kinetics. The presence of long aliphatic chains (2amino-16C-TMS-Xero) does not further seem to affect the CO_2_ diffusion kinetics.

## 4 Conclusions

The silica gels with hybrid grafting of amine, aliphatic, and trimethylsilyl groups presented in this study exhibit considerable potential as direct air capture (DAC) sorbents, offering high CO_2_/H_2_O selectivity and potentially outperforming traditional amine-functionalized resins with hydrophobic backbones, although further material optimization (e.g., CO_2_ sorption capacity) and analysis on possible working performance are required.

Due to significant water sorption and capillary condensation occurring at the high relative humidity typical for working conditions of DAC, hydrophilic silica gels are not a realistic option for real-world implementation. Single-component sorption measurements of CO_2_ under dry conditions and breakthrough measurements of wet CO_2_ at low humidity are insufficient to fully characterize potential DAC sorbents. They cannot accurately predict the sorption behavior under realistic long-exposure DAC conditions. For that purpose, a water saturation step at high-relative humidity should be carried out before the CO_2_ breakthrough experiments.

Hydrophobization with trimethylsilyl groups allows the maintenance of a constant CO_2_ uptake capacity and kinetic constant in the sample saturated with water below 80% RH. However, the presence of trimethylsilyl groups reduces the CO_2_ diffusion under dry conditions compared to samples with only amine groups grafted. Grafting long aliphatic chains does not slow down CO_2_ diffusion further and effectively prevents capillary condensation at high humidity, possibly by shielding the hydrophilic amine groups from bulk water and maintaining the hydrophobicity of the pore surfaces.

Given the combinatorial diversity of amorphous solids, this study explores only a limited number of the parameters that describe their complexity. A broader exploration of aliphatic chain lengths, grafting density, particle size, surface area, and pore size distribution may reveal new phenomena and suggest additional optimization strategies. Among the results reported in this study, the successful application of ambient pressure drying without a significant loss of selectivity and the low-cost silica matrix is particularly promising. However, further characterization and evaluation of the samples presented in this work are necessary for DAC applications, including, but not limited to, temperature-dependent adsorption behavior, heats of sorption, and aging studies. Silica gels have already been produced on a mass scale. Therefore, scaling up the synthesis and functionalization methods for these silica gels is unlikely to pose an insurmountable challenge.

## Supplementary Material



## Notes

Analysis of breakthrough measurement uncertainty, additional experimental details, CO_2_ adsorption isotherms on 2xConc.-2amino-TMS-Xero at 0, 25, and 50 °C, ^13^C spectra predictions for solid-state nuclear magnetic resonance, details on breakthrough modeling, scanning electron microscopy photographs, optical microscopy photographs and particle size distribution of the sieved gel used in breakthrough experiments, and water contact angle measurements (PDF)

## References

[1] Lynas M., Houlton B. Z., Perry S. (2021). Greater than 99% Consensus on Human
Caused Climate Change in the Peer-Reviewed Scientific Literature. Environ. Res. Lett..

[2] Myhre, G. ; Shindell, D. ; Bréon, F.-M. ; Collins, W. ; Fuglestvedt, J. ; Huang, J. ; Koch, D. ; Lamarque, J.-F. ; Lee, D. ; Mendoza, B. ; Nakajima, T. ; Robock, A. ; Stephens, G. ; Takemura, T. ; Zhang, H. Anthropogenic and Natural Radiative Forcing. In Climate Change 2013—The Physical Science Basis; Cambridge University Press, 2013; Vol. 6, pp 659–740, 10.1017/CBO9781107415324.018.

[3] World Meteorological Organization . State of the Global Climate 2023; United Nations, 2024; 10.18356/9789263113474.

[4] Copernicus Climate Change Service. Copernicus: 2024 Is the First Year with All Days over 1.5°C above Pre-industrial Level. https://climate.copernicus.eu/copernicus-2024-first-year-exceed-15degc-above-pre-industrial-level.

[5] Le
Quéré C., Raupach M. R., Canadell J. G., Marland G., Bopp L., Ciais P., Conway T. J., Doney S. C., Feely R. A., Foster P., Friedlingstein P., Gurney K., Houghton R. A., House J. I., Huntingford C., Levy P. E., Lomas M. R., Majkut J., Metzl N., Ometto J. P., Peters G. P., Prentice I. C., Randerson J. T., Running S. W., Sarmiento J. L., Schuster U., Sitch S., Takahashi T., Viovy N., van der Werf G. R., Woodward F. I. (2009). Trends in the Sources and Sinks of Carbon Dioxide. Nat. Geosci..

[6] El Sherif, D. ; Knox, J. C. International Space Station Carbon Dioxide Removal Assembly (ISS CDRA) Concepts and Advancements. SAE Technical Paper 2005-01-2892; SAE International, 2005; 10.4271/2005-01-2892.

[7] Buck H. J., Carton W., Lund J. F., Markusson N. (2023). Why Residual
Emissions Matter Right Now. Nat. Clim. Chang..

[8] Pathak, M. ; Slade, R. ; Shukla, P. R. ; Skea, J. ; Pichs-Madruga, R. ; Ürge-Vorsatz, D. Technical Summary. In Climate Change 2022—Mitigation of Climate Change; Cambridge University Press, 2023; pp 51–148, 10.1017/9781009157926.002.

[9] Ozkan M., Nayak S. P., Ruiz A. D., Jiang W. (2022). Current Status and
Pillars of Direct Air Capture Technologies. iScience.

[10] Herzog, H. Direct Air Capture. In Greenhouse Gas Removal Technologies; The Royal Society of Chemistry, 2022; pp 115–137, 10.1039/9781839165245-00115.

[11] Agnolucci P., Fischer C., Heine D., Montes de Oca Leon M., Pryor J., Patroni K., Hallegatte S. (2024). Measuring
Total Carbon Pricing. World Bank Res. Obs..

[12] Wang Y., Li G. K. (2025). The Impact of Co-Adsorbed Water on Energy Consumption and CO_2_ Productivity in Direct Air Capture Systems. Sep. Purif. Technol..

[13] Satyapal S., Filburn T., Trela J., Strange J. (2001). Performance
and Properties
of a Solid Amine Sorbent for Carbon Dioxide Removal in Space Life
Support Applications. Energy Fuels.

[14] Low M. Y., Barton L. V., Pini R., Petit C. (2023). Analytical Review of
the Current State of Knowledge of Adsorption Materials and Processes
for Direct Air Capture. Chem. Eng. Res. Des..

[15] New M., Lister D., Hulme M., Makin I. (2002). A High-Resolution Data
Set of Surface Climate over Global Land Areas. Clim. Res..

[16] Findley J. M., Sholl D. S. (2021). Computational Screening of MOFs and Zeolites for Direct
Air Capture of Carbon Dioxide under Humid Conditions. J. Phys. Chem. C.

[17] Bose S., Sengupta D., Rayder T. M., Wang X., Kirlikovali K. O., Sekizkardes A. K., Islamoglu T., Farha O. K. (2024). Challenges and Opportunities:
Metal–Organic Frameworks for Direct Air Capture. Adv. Funct. Mater..

[18] Leonzio G., Fennell P. S., Shah N. (2022). A Comparative Study of Different
Sorbents in the Context of Direct Air Capture (DAC): Evaluation of
Key Performance Indicators and Comparisons. Appl. Sci..

[19] Wurzbacher J. A., Gebald C., Piatkowski N., Steinfeld A. (2012). Concurrent
Separation of CO_2_ and H_2_O from Air by a Temperature-Vacuum
Swing Adsorption/Desorption Cycle. Environ.
Sci. Technol..

[20] Goeppert A., Meth S., Prakash G. K. S., Olah G. A. (2010). Nanostructured Silica
as a Support for Regenerable High-Capacity Organoamine-Based CO_2_ Sorbents. Energy Environ. Sci..

[21] Sanz-Pérez E. S., Olivares-Marín M., Arencibia A., Sanz R., Calleja G., Maroto-Valer M. M. (2013). CO_2_ Adsorption Performance of Amino-Functionalized SBA-15 under
Post-Combustion Conditions. Int. J. Greenh.
Gas Control.

[22] Shi X., Xiao H., Azarabadi H., Song J., Wu X., Chen X., Lackner K. S. (2020). Sorbents for the Direct Capture of
CO2 from Ambient Air. Angew. Chemie Int. Ed..

[23] Meng Y., Jiang J., Gao Y., Yan F., Liu N., Aihemaiti A. (2018). Comprehensive Study of CO_2_ Capture Performance
under a Wide Temperature Range Using Polyethyleneimine-Modified Adsorbents. J. CO2 Util..

[24] Elfving J., Kauppinen J., Jegoroff M., Ruuskanen V., Järvinen L., Sainio T. (2021). Experimental Comparison of Regeneration
Methods for CO_2_ Concentration from Air Using Amine-Based
Adsorbent. Chem. Eng. J..

[25] Said R. B., Kolle J. M., Essalah K., Tangour B., Sayari A. (2020). A Unified
Approach to CO_2_-Amine Reaction Mechanisms. ACS Omega.

[26] Young J., García-Díez E., Garcia S., Van Der
Spek M. (2021). The Impact of Binary Water-CO_2_ isotherm Models on the
Optimal Performance of Sorbent-Based Direct Air Capture Processes. Energy Environ. Sci..

[27] Gebald C., Wurzbacher J. A., Borgschulte A., Zimmermann T., Steinfeld A. (2014). Single-Component
and Binary CO_2_ and H_2_O Adsorption of Amine-Functionalized
Cellulose. Environ. Sci. Technol..

[28] Elfving J., Sainio T. (2021). Kinetic Approach to
Modelling CO_2_ Adsorption
from Humid Air Using Amine-Functionalized Resin: Equilibrium Isotherms
and Column Dynamics. Chem. Eng. Sci..

[29] Thommes M., Kaneko K., Neimark A. V., Olivier J. P., Rodriguez-Reinoso F., Rouquerol J., Sing K. S. W. (2015). Physisorption of Gases, with Special
Reference to the Evaluation of Surface Area and Pore Size Distribution
(IUPAC Technical Report). Pure Appl. Chem..

[30] Hüsing N., Schubert U. (1998). Aerogels—Airy Materials: Chemistry, Structure,
and Properties. Angew. Chemie Int. Ed..

[31] Li Z., Zhao S., Koebel M. M., Malfait W. J. (2020). Silica Aerogels
with Tailored Chemical Functionality. Mater.
Des..

[32] Malfait W. J., Zhao S., Verel R., Iswar S., Rentsch D., Fener R., Zhang Y., Milow B., Koebel M. M. (2015). Surface
Chemistry of Hydrophobic Silica Aerogels. Chem.
Mater..

[33] Xu B., Zhang Q. (2021). Preparation and Properties of Hydrophobically Modified
Nano-SiO2
with Hexadecyltrimethoxysilane. ACS Omega.

[34] Quang D. V., Hatton T. A., Abu-Zahra M. R. M. (2016). Thermally Stable Amine-Grafted Adsorbent
Prepared by Impregnating 3-Aminopropyltriethoxysilane on Mesoporous
Silica for CO_2_ Capture. Ind. Eng.
Chem. Res..

[35] Wadi B., Golmakani A., Manovic V., Nabavi S. A. (2021). Evaluation
of Moderately
Grafted Primary, Diamine, and Triamine Sorbents for CO2 Adsorption
from Ambient Air: Balancing Kinetics and Capacity under Humid Conditions. Ind. Eng. Chem. Res..

[36] Wörmeyer K., Alnaief M., Smirnova I. (2012). Amino Functionalised
Silica-Aerogels
for CO_2_-Adsorption at Low Partial Pressure. Adsorption.

[37] Wörmeyer K., Smirnova I. (2013). Adsorption of CO_2_, Moisture and Ethanol
at Low Partial Pressure Using Aminofunctionalised Silica Aerogels. Chem. Eng. J..

[38] Cui S., Cheng W., Shen X., Fan M., Russell A. T., Wu Z., Yi X. (2011). Mesoporous Amine-Modified SiO_2_ Aerogel:
A Potential CO_2_ Sorbent. Energy Environ.
Sci..

[39] Begag R., Krutka H., Dong W., Mihalcik D., Rhine W., Gould G., Baldic J., Nahass P. (2013). Superhydrophobic Amine
Functionalized Aerogels as Sorbents for CO2 Capture. Greenh. Gases Sci. Technol..

[40] Jiang X., Kong Y., Zhao Z., Shen X. (2020). Spherical Amine Grafted
Silica Aerogels for CO_2_ capture. RSC Adv..

[41] Wörmeyer K., Smirnova I. (2014). Breakthrough Measurements of CO_2_ through
Aminofunctionalised Aerogel Adsorbent at Low Partial Pressure: Experiment
and Modeling. Microporous Mesoporous Mater..

[42] Keshavarz L., Ghaani M. R., MacElroy J. M. D., English N. J. (2021). A Comprehensive
Review on the Application of Aerogels in CO_2_-Adsorption:
Materials and Characterisation. Chem. Eng. J..

[43] Maleki, H. ; Hüsing, N. Aerogels as Promising Materials for Environmental Remediation—A Broad Insight into the Environmental Pollutants Removal through Adsorption and (Photo)­Catalytic Processes. In New Polymer Nanocomposites for Environmental Remediation; Elsevier, 2018; pp 389–436, 10.1016/B978-0-12-811033-1.00016-0.

[44] Pajonk G. M., Elaloui E., Achard P., Chevalier B., Chevalier J.-L., Durant M. (1995). Physical Properties of Silica Gels
and Aerogels Prepared with New Polymeric Precursors. J. Non. Cryst. Solids.

[45] Brunauer S., Emmett P. H., Teller E. (1938). Adsorption of Gases
in Multimolecular
Layers. J. Am. Chem. Soc..

[46] Rouquerol J., Llewellyn P., Rouquerol F. (2007). Is the Bet Equation Applicable to
Microporous Adsorbents?. Stud. Surf. Sci. Catal..

[47] Barrett E. P., Joyner L. G., Halenda P. P. (1951). The Determination
of Pore Volume
and Area Distributions in Porous Substances. I. Computations from
Nitrogen Isotherms. J. Am. Chem. Soc..

[48] de
Boer J. H., Lippens B. C., Linsen B. G., Broekhoff J. C. P., van den Heuvel A., Osinga T. J. (1966). Thet-Curve of Multimolecular
N2-Adsorption. J. Colloid Interface Sci..

[49] Malfait W. J., Verel R., Koebel M. M. (2014). Hydrophobization of Silica Aerogels:
Insights from Quantitative Solid-State NMR Spectroscopy. J. Phys. Chem. C.

[50] Hall B. D., Crotwell A. M., Kitzis D. R., Mefford T., Miller B. R., Schibig M. F., Tans P. P. (2021). Revision
of the World Meteorological
Organization Global Atmosphere Watch (WMO/GAW) CO_2_ Calibration
Scale. Atmos. Meas. Technol..

[51] Wilkins N. S., Sawada J. A., Rajendran A. (2022). Quantitative
Microscale Dynamic Column
Breakthrough Apparatus for Measurement of Unary and Binary Adsorption
Equilibria on Milligram Quantities of Adsorbents. Ind. Eng. Chem. Res..

[52] Sharma S., Balestra S. R. G., Baur R., Agarwal U., Zuidema E., Rigutto M. S., Calero S., Vlugt T. J. H., Dubbeldam D. (2023). RUPTURA: Simulation
Code for Breakthrough, Ideal Adsorption Solution Theory Computations,
and Fitting of Isotherm Models. Mol. Simul..

[53] Li C., Zhang G., Lin L., Wu T., Brunner S., Galmarini S., Bi J., Malfait W. J., Zhao S., Ostrikov K. (2023). Silica Aerogels: From Materials Research
to Industrial
Applications. Int. Mater. Rev..

[54] Sivaraman D., Zhao S., Iswar S., Lattuada M., Malfait W. J. (2021). Aerogel
Spring-Back Correlates with Strain Recovery: Effect of Silica Concentration
and Aging. Adv. Eng. Mater..

[55] Reichenauer, G. Structural Characterization of Aerogels. In Aerogels Handbook; Springer New York: New York, NY, 2011; Vol. 27, pp 449–498, 10.1007/978-1-4419-7589-8_21.

[56] Anderson, A. M. ; Carroll, M. K. Hydrophobic Silica Aerogels: Review of Synthesis, Properties and Applications. In Aerogels Handbook; Springer New York: New York, NY, 2011; pp 47–77, 10.1007/978-1-4419-7589-8_3.

[57] Kocherbitov V., Alfredsson V. (2007). Hydration of MCM-41 Studied by Sorption Calorimetry. J. Phys. Chem. C.

[58] Wallace S., Hench L. L. (1994). Structural Analysis
of Water Adsorbed in Silica Gel. J. Sol-Gel
Sci. Technol..

[59] Li K., Kress J. D., Mebane D. S. (2016). The Mechanism of CO_2_ Adsorption
under Dry and Humid Conditions in Mesoporous Silica-Supported Amine
Sorbents. J. Phys. Chem. C.

[60] Alesi W. R., Kitchin J. R. (2012). Evaluation of a
Primary Amine-Functionalized Ion-Exchange
Resin for CO_2_ Capture. Ind. Eng.
Chem. Res..

[61] Veneman R., Zhao W., Li Z., Cai N., Brilman D. W. F. (2014). Adsorption
of CO_2_ and H_2_O on Supported Amine Sorbents. Energy Procedia.

[62] Rouquerol, F. ; Rouquerol, J. ; Sing, K. S. W. ; Llewellyn, P. ; Maurin, G. Adsorption by Powders and Porous Solids. Principles, Methodology and Applications, 2nd ed.; Academic Press, 2014.

[63] Ahmadalinezhad A., Sayari A. (2014). Oxidative
Degradation of Silica-Supported Polyethylenimine
for CO_2_ Adsorption: Insights into the Nature of Deactivated
Species. Phys. Chem. Chem. Phys..

[64] Choi S., Gray M. L., Jones C. W. (2011). Amine-Tethered
Solid Adsorbents Coupling
High Adsorption Capacity and Regenerability for CO_2_ Capture
from Ambient Air. ChemSusChem.

[65] Chahbani M. H., Tondeur D. (2001). Pressure Drop in Fixed-Bed
Adsorbers. Chem. Eng. J..

[66] Zhang W., Shan Y., Seidel-Morgenstern A. (2006). Breakthrough
curves and elution profiles
of single solutes in case of adsorption isotherms with two inflection
points. J. Chromatogr. A.

[67] McCool B. A., DeSisto W. J. (2005). Amino-Functionalized
Silica Membranes for Enhanced
Carbon Dioxide Permeation. Adv. Funct. Mater..

