# Supplementary material for: Hydrophobic Silica Gels and Aerogels for Direct Air Capture: Hybrid Grafting To Suppress Water Uptake and Capillary Condensation
Source: ACS Appl Mater Interfaces. 2025 Jul 17;17(30):42954–68. doi: 10.1021/acsami.5c04491 (PMC12314860; doi:10.1021/acsami.5c04491)
Supplement: Supplementary file 1 [file am5c04491_si_001.pdf]

Supporting Information

Hydrophobic Silica Gels and Aerogels for Direct Air Capture: Hybrid Grafting to Suppress Water Uptake and Capillary Condensation

Paweł P. Ziemiański[1,2,3*], Mercy Cherotich[1], Ilia Sadykov[1], Chaima Mansour[1], Ivan Lunati[2], Marco Ranocchiari[3], Wim J. Malfait[1], Sandra Galmarini[1*]

[1] Building Energy Materials and Components, Empa, 8600 Dübendorf, Switzerland.

[2] Laboratory for Computational Engineering, Empa, 8600 Dübendorf, Switzerland

[3] PSI Center for Energy and Environmental Sciences (CEE), 5232 Villigen PSI, Switzerland

Corresponding authors: Paweł. P. Ziemiański, pawel.ziemianski@empa.ch, Sandra Galmarini, sandra.galmarini@empa.ch

1 Analysis of breakthrough measurement uncertainty

An error analysis was performed to evaluate the significance of the calculated CO_2 loadings, $q*_{ads}$, calculated for the equilibrium time t* (and t**, bypassing the column) using equation 1 and adapting the mass balance equation given by Wilkins et al., (2022)[1]. As the contribution of V_D is negligible due to the dilute CO_2 concentrations, and because the concentration at the outlet, C_{out}, for equilibrium time t* is measured N times, the CO_2 loadings can be approximated as a sum (eq. 1),

$$\begin{aligned} q*_{ads} &= \frac{F}{m_s}\left[\int_{t_0^{ads}}^{t_0^{ads}+t*}(C_{in}-C_{out})\,dt - \int_{t_0^{bypass}}^{t_0^{bypass}+t**}(C_{in}-C_{out})\,dt\right] - \frac{V_D * C_{in}}{m_s} \\ &\approx \frac{F}{m_s}\left(\sum_{i=n_0^{ads}}^{n_0^{ads}+N_{ads}}\left[(C_{in}-C_{out_i})\Delta t\right] - \sum_{j=n_0^{bypass}}^{n_0^{bypass}+N_{bypass}}\left[(C_{in}-C_{out_j})\Delta t\right]\right) \\ &= \frac{F\Delta t}{m_s}\left((N_{ads}-N_{bypass})C_{in} - \sum_{i=n_0^{ads}}^{n_0^{ads}+N_{ads}} C_{out_i} + \sum_{j=n_0^{bypass}}^{n_0^{bypass}+N_{bypass}} C_{out_j})\right) \\ &= \hat{q}*_{ads} \end{aligned} \tag{1}$$

Where $\Delta t = \frac{t*}{N_{ads}} = \frac{t**}{N_{bypass}}$ is the measurement interval of c_{out}.

Five measured variables contribute to the uncertainty of the adsorbed C_2 calculated at equilibrium time t*: the volumetric flow, F; the dead volume, V_D; the sorbent mass, m_s; and the molar concentration of the gas at the inlet and outlet, C_{in} and C_{out}, respectively. Assuming that all errors are independent, the total error of the measurement at time t* can be estimated as [2],

$$\hat{\sigma}_{\hat{q}*_{ads}} = \sqrt{(\frac{\partial \hat{q}*_{ads}}{\partial F}\sigma_F)^2 + (\frac{\partial \hat{q}*_{ads}}{\partial m_s}\sigma_{m_s})^2 + (\frac{\partial \hat{q}*_{ads}}{\partial C_{in}}\sigma_{C_{in}})^2 + \sum_i^{N_{ads}}(\frac{\partial \hat{q}*_{ads}}{\partial C_{out_i}}\sigma_{C_{out}})^2 + \sum_j^{N_{bypass}}(\frac{\partial \hat{q}*_{ads}}{\partial C_{out_j}}\sigma_{C_{out}})^2}, \tag{2}$$

where the partial derivatives are,

$$\frac{\partial \hat{q}*_{ads}}{\partial F} = \frac{\Delta t}{m_s}\left((N_{ads}-N_{bypass})C_{in} - \sum_{i=n_0^{ads}}^{n_0^{ads}+N_{ads}} C_{out_i} + \sum_{j=n_0^{bypass}}^{n_0^{bypass}+N_{bypass}} C_{out_j})\right) = \frac{\hat{q}*_{ads}}{F}, \tag{3}$$

$$\frac{\partial \hat{q}*_{ads}}{\partial m_s} = -\frac{F\Delta t}{m_s^2}\left((N_{ads}-N_{bypass})C_{in} - \sum_{i=n_0^{ads}}^{n_0^{ads}+N_{ads}} C_{out_i} + \sum_{j=n_0^{bypass}}^{n_0^{bypass}+N_{bypass}} C_{out_j})\right) = -\frac{\hat{q}*_{ads}}{m_s}, \tag{4}$$

$$\frac{\partial \hat{q}*_{ads}}{\partial C_{in}} = \frac{F\Delta t}{m_s}(N_{ads}-N_{bypass}) = \frac{F}{m_s}(t^* - t^{**}), \tag{5}$$

$$\frac{\partial \hat{q}*_{ads}}{\partial C_{out_i}} = -\frac{F\Delta t}{m_s} = -\frac{Ft^*}{N_{ads}m_s}. \tag{6}$$

and

$$\frac{\partial \hat{q}*_{ads}}{\partial C_{out_j}} = \frac{F\Delta t}{m_s} = \frac{Ft^{**}}{N_{bypass}m_s}. \tag{7}$$

By inserting these equations into Eq. 2, we obtain

$$\hat{\sigma}_{\hat{q}*_{ads}} = \sqrt{(\frac{\hat{q}*_{ads}}{F}\sigma_F)^2 + (\frac{\hat{q}*_{ads}}{m_s}\sigma_{m_s})^2 + (\frac{F}{m_s}(t^* - t^{**})\sigma_{C_{in}})^2 + (\frac{t^{*2}}{N_{ads}} + \frac{t^{**2}}{N_{bypass}})(\frac{F}{m_s}\sigma_{C_{out}})^2} \tag{8}$$

Using Equation 10, the same analysis is performed to determine the uncertainty in the amount of desorbed CO_2 at equilibrium time t*,

$$\hat{q}*_{des} = \frac{F\Delta t}{m_s}\left(-\sum_{i=n_0^{des}}^{n_0^{des}+N_{des}} C_{out_i})\right) \tag{9}$$

where the bypass term is omitted during desorption. Hence, we obtain

$$\hat{\sigma}_{\hat{q}*_{des}} = \sqrt{(\frac{\hat{q}*_{des}}{F}\sigma_F)^2 + (\frac{\hat{q}*_{des}}{m_s}\sigma_{m_s})^2 + (t^*\Delta t)(\frac{F}{m_s}\sigma_{C_{out}})^2} \tag{10}$$

2 Skeletal and envelope density

The skeletal density, referring to the density of the solid framework, was measured by the AccuPyc II 1340 Helium Pycnometer (Micromeritics). Prior to the measurement, samples were dried in a vacuum oven at 100°C for 24 hours. Then, the samples were quickly transferred to a 1 cm^3 insert and sealed in the device. The density values were recorded when an equilibration rate of 0.0345 kPa/min was achieved. The envelope density measurements were taken with Geopyc 1360 (Micromeritics). Envelope density refers to the overall density of a material, accounting for both the solid framework and any internal porosity within the particles but excluding interparticle voids. After drying (the same conditions as for skeletal density measurements), samples were weighted and loaded into a measurement chamber to fill 25% of its total volume (volume of Sample + volume of Dry Flo sand). The skeletal and envelope densities were used to calculate bed porosity for breakthrough simulations.

3 CO_2 sorption at various temperatures

The CO_2 adsorption isotherms were measured using ASAP 2020. 100 mg of each sample was loaded into the sample tube and activated under high vacuum (5 µbar) at 100 °C for 20h. The temperature was thermostated during the experiments using a CORIO CD-200F thermostat at 0, 25, and 50 °C, with stability ±0.03°C. The resulting isotherms are presented in Fig. 1

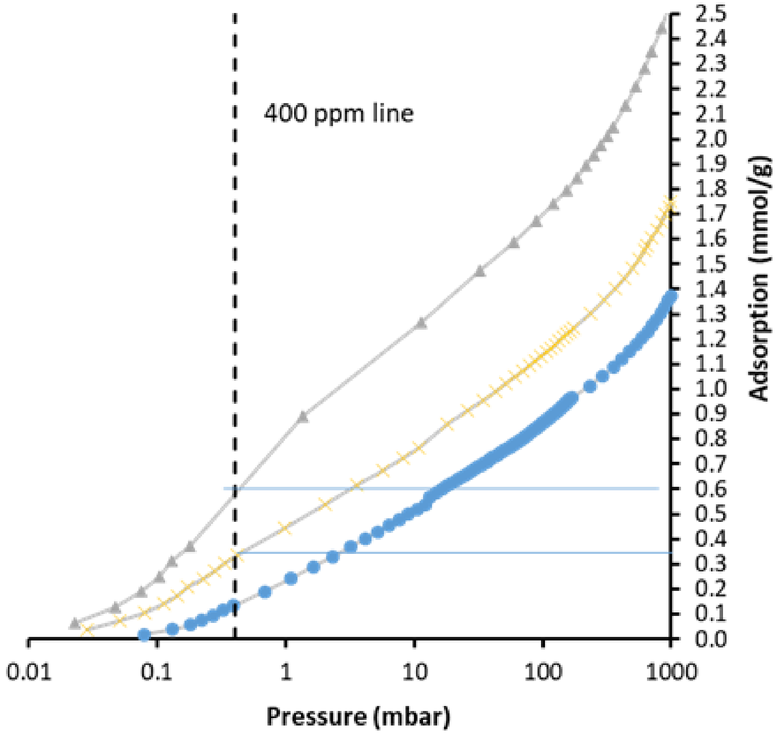

Figure S1: Single component CO_2 adsorption isotherms measured on silica gel (2Conc.-2amino-TMS-Xero) at 0 (grey), 25 (yellow), and 50°C (blue). 400 ppm line corresponds to 0.4 mbar.

4 The ^{13}C spectra predictions for solid state nuclear magnetic resonance

The ^{13}C NMR spectra were predicted with ChemDraw® 23.0 software (Revvity Signal Software, Inc.) and are shown in Figures 2 and 3.

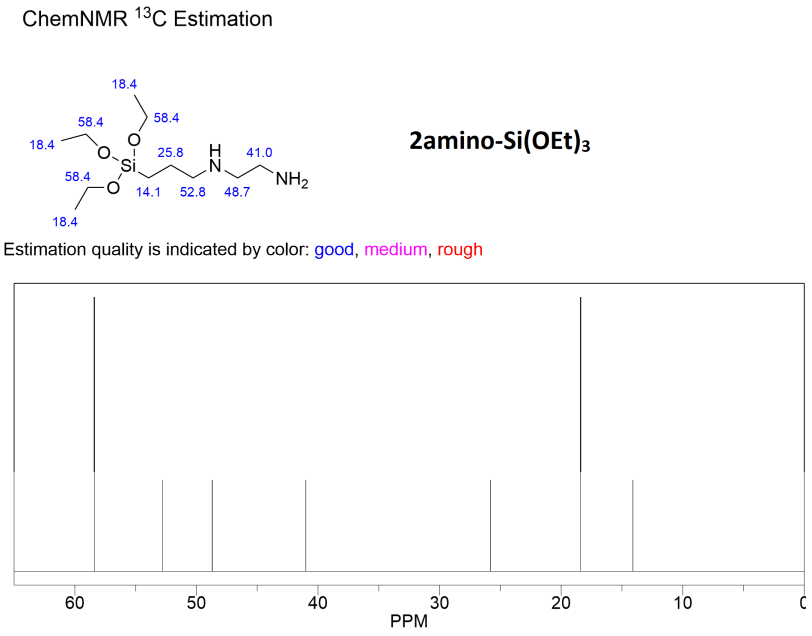

Figure S2: The ^{13}C spectra predictions for (N-3-triethoxysilyl)propylethylenediamine made with ChemDraw 23.0 software.

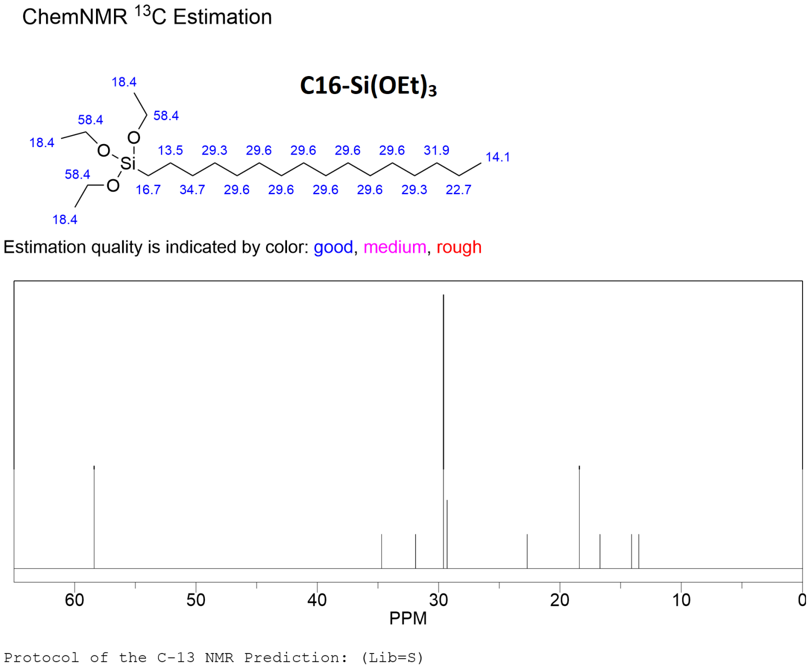

Figure S3: The ^{13}C spectra predictions for hexadecyltrimthoxysilane made with ChemDraw 23.0 software.

5 Breakthrough modeling

Experimental breakthrough curves were modeled in RUPTURA software [3] by varying first-order kinetic constants k (linear driving force) that correspond to the following equation of the linear driving force:

$$\frac{\partial q_{ads}^{bead}}{\partial t} = k(q_{ads}^{*} - q_{ads}^{bead}) \tag{11}$$

, where q_{ads}^{bead} is the average CO$_2$ uptake [mol/kg] inside the bead, q_{ads}^{*} is the equilibrium CO_2

uptake [mol/kg] that corresponds to the CO_2 pressure in the gas stream outside of the sorbent CO_2, k is the Linear Driving Force kinetic constant.

The kinetic constant k was fitted by minimizing the root mean square (RMSE) difference between the experimental and modeled breakthrough up to the CO_2 concentrations of 380 ppm:

$$RMSE = \sqrt{\frac{\sum_0^N (y_i - y)^2}{N^2}} \tag{12}$$

, where N is the number of points on the breakthrough curve.

A linear (Henry) isotherm was used:

$$q_{ads} = H_{CO2} \cdot p_{CO_2} \tag{13}$$

to approximate the uptake of the samples during breakthrough, H_{CO2} values $(mol\ kg^{-1}Pa^{-1})$ were obtained by dividing the total uptake during breakthrough (q^*_{ads}) $(mol\ kg^{-1})$ by the final CO_2 pressure (p_{400ppm}; 39.1 Pa).

$$H_{CO2} = \frac{q^*_{ads}}{p_{400ppm}} \tag{14}$$

The resulting values are shown in the main text in Table 3.

Since CO_2 adsorption was described with the Henry isotherm (equation 14), the linear driving force equation used in RUPTURA is equivalent to a mass diffusion term:

$$\frac{\partial p^{pore}_{CO_2}}{\partial t} = k(p_{CO_2} - p^{pore}_{CO_2}) \tag{15}$$

, where $p^{pore}_{CO_2}$ is the average CO_2 pressure inside of the spherical particle (sorbent bead), p_{CO_2} is the pressure of CO_2 in the gas stream outside of the sorbent pores, and k is the kinetic coefficient inversely proportional to the mass transport resistance.

Mass transport resistance for CO_2 can also be calculated using an analytical solution to the steady-state diffusion through the sphere described previously [4, 5]:

$$R^{mass}_{CO_2} = \frac{3}{4 \cdot \pi^3 \cdot r_p \cdot D_{CO_2} \cdot \epsilon_{bead}} \tag{16}$$

, where ϵ_{bead} is the porosity of the spherical sorbent beads, D_{CO_2} is the effective CO_2 macroscopic diffusion coefficient, and r_p is the radius of the bead.

In this case, a change in the internal pressure of CO_2 inside macropores $p^{pore}_{CO_2}$ is described as:

$$V_p \cdot \frac{\partial p^{pore}_{CO_2}}{\partial t} = \frac{(p_{CO_2} - p^{pore}_{CO_2})}{R^{mass}_{CO_2}} - V_{env} \cdot \frac{\partial q_{ads}}{\partial t} \cdot \rho_{env} \cdot RT \tag{17}$$

, where V_p is the pore volume inside the spherical bead of sorbent, R is the ideal gas constant, V_{env} and ρ_{env} are envelope volume and density, respectively, and T is temperature. Using the analytical mass resistance of a sphere as well as the Henry isotherm, we can simplify Equation 17:

$$\frac{\partial p^{pore}_{CO_2}}{\partial t}(1 + \frac{H_{CO2}\rho_{env}RT}{\epsilon_{sorbent}}) = \frac{(p_{CO_2} - p^{pore}_{CO_2})\pi^2 D_{CO_2}}{r_p^2} \tag{18}$$

, porosity of the sorbent bead $\epsilon_{sorbent}$ is calculated as $\epsilon_{bead} = \frac{V_p}{V_{env}}$.

Using the kinetic constant k fitted to experiments, the diffusion coefficient can finally be calculated as:

$$k = \frac{\pi^2 D_{CO_2}}{r_p^2} \frac{\epsilon_{sorbent}}{(\epsilon_{sorbent} + H_{CO2}\rho_{env}RT)} \tag{19}$$

$$D_{CO_2} = \frac{k r_p^2(\epsilon_{sorbent} + H_{CO2}\rho_{env}RT)}{\epsilon_{sorbent}\pi^2} \tag{20}$$

The radius of the spherical particle r_p was taken to be equal to 200 μm for functionalized silica.

Experimental breakthrough parameters used to model the breakthrough curves are summarized in Tables 1 and 2. We used the formula

$$\epsilon_{bead} = 1 - \frac{\rho_{env}}{\rho_{skel}} \tag{21}$$

to calculate the porosity of the spherical sorbent beads. Here ρ_{skel} is the skeletal density of the sorbent. All porosities, skeletal, and envelope densities are summarized in Tables S1 and S2 below. The porosity of the packed bed was calculated using an equation below from the mass and the volume of the sorbent bed: m and V_{bed}.

$$\epsilon_{bed} = 1 - \frac{m}{\rho_{env} \cdot V_{bed}} \tag{22}$$

**Sample name**	**Bed lenght**	**Bed porosity**	**Inlet flow**	**Sample mass**
	**cm**		**sccm**	**mg**
2amino-Xero	2.2	0.12	52.5	184
2amino-16C-TMS-Xero	4.0	0.13	52.5	190
2xConc.-2amino-TMS-Xero	2.5	0.12	52.5	128

Table S1: Breakthrough simulation parameters for RUPTURA software [3].

**Sample name**	**Envelope density**	**Skeletal density**	**Spherical bead porosity**
	$kg\ m^{-3}$	$kg\ m^{-3}$	
2amino-Xero	757	1645	0.54
2amino-16C-TMS-Xero	513	1432	0.64
2xConc.-2amino-TMS-Xero	686	1658	0.59

Table S2: Breakthrough simulation parameters for RUPTURA software [3].

The estimated effective diffusion coefficients were calculated from the breakthrough kinetic constants (k) using an analytical solution to the mass transport through the spherical particle, as shown Eq. 20. The results can be found in Table S3.

The presented diffusion coefficient (D_{CO2}) values are semi-quantitative at best and should be interpreted with a high degree of caution. They are included solely for comparison with literature data, which itself shows significant variability. This highlights the inherent uncertainty in calculating diffusion coefficients from mass transfer coefficients and reinforces the need for careful interpretation when comparing diffusion-related parameters across studies.

2xConc.-2amino-TMS-Xero and 2amino-16C-TMS-Xero samples showed similar D_{CO2} on the order of 10^{-7} m^2/s under dry and humid conditions. The diffusion coefficient corresponding to the Knudsen diffusion regime in pores of diameter 5-10 nm (similar to the pore size range for xerogels; Tab. 1) for diluted CO2 at 25°C and atmospheric pressure are in the range of 10^{-7} – 10^{-6} m^2/s [6].

These values match the diffusion coefficient calculated for silica xerogels, except for dry 2amino-Xero, which is significantly higher (Tab. 3).

As it was previously demonstrated [7], the diffusion coefficients calculated for hydrophobized silica gels are similar to the effective CO_2 diffusivity at atmospheric pressure for silica gel without functionalization (10^{-6}–10^{-7} m^2 s^{-1}). However, the hydrophobized samples (2-amino-16C-TMS-Xero and 2xConc.-2-amino-TMS-Xero) have faster kinetics than the amino-functionalized silicas that were previously studied. For example, silica gel modified with N1-(3-trimethoxysilylpropyl)diethylenetriamine displayed an effective CO_2 diffusivity of 1.1×10^{-10} m^2 s^{-1} for 400 μm-diameter particles [8], while APTES-modified silica with an internal pore size of 4.7 nm had an effective CO_2 diffusivity of 4.92×10^{-11} m^2 s^{-1} [9]. The calculated diffusion coefficients are comparable to those reported by [10], who found a value of 3.5×10^{-6} m^2 s^{-1} for CO_2 sorption on silica gels with grafted mono- and triamines under 30% RH; however, the authors could not reproduce the shape of the breakthrough curve.

Sample / State	Kinetic constant k (s^{-1})	D_{CO2} (m^2/s)	H_{CO2} (mol kg^{-1} Pa^{-1})	RMSE (ppm)
2amino-Xero / dry	0.10	1.5×10^{-5}*	0.0118	0.4
2amino-Xero / water-saturated	N.A.	N.A.	0.0017	0.6
2amino-16C-TMS-Xero / dry	0.01	3.2×10^{-7}	0.0084	0.3
2amino-16C-TMS-Xero / water-saturated	0.01	5.3×10^{-7}	0.0078	0.2
2xConc.-2amino-TMS-Xero / dry	0.01	6.6×10^{-7}	0.0118	0.2
2xConc.-2amino-TMS-Xero / water-saturated	0.01	4.0×10^{-7}	0.0105	0.3

Table S3: Breakthrough simulation results for dry and humid flows and Henry coefficients used. * D_{CO_2} for the 2amino-Xero sample in dry conditions corresponds to the diffusion of CO_2 in air at 400 ppm, 1 bar, and 25 °C. Due to high fitting error, constants for water-saturated 2amino-Xero are not reported.

Dimensionless time derived from the gas velocity (ν $[m\ s^{-1}]$), bed length (L $[m]$), and bed porosity (ϵ) is used to better compare experimental breakthrough curves [3]. Such a comparison makes it possible to compare these samples on the same scale, with equalized effects from porosity and bed length. They confirm that hydrophobized samples perform much better under humid conditions than non-hydrophobized ones.

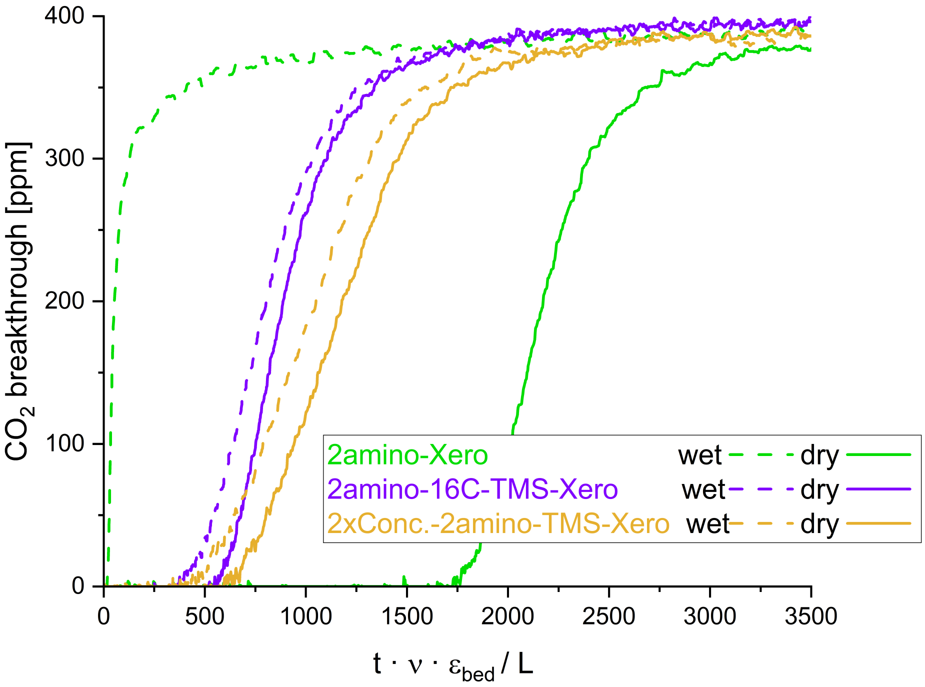

Figure S4: Breakthrough curves of the modelled samples plotter against dimensionless time.

6 Scanning Electron Microscopy

The morphology of the samples was analyzed using a Zeiss GeminiSEM 460 scanning electron microscope (SEM). Imaging was performed in high vacuum mode (less than 10^{-2} Pa) using the InLens secondary electron (SE) detector at an accelerating voltage of 2 kV.

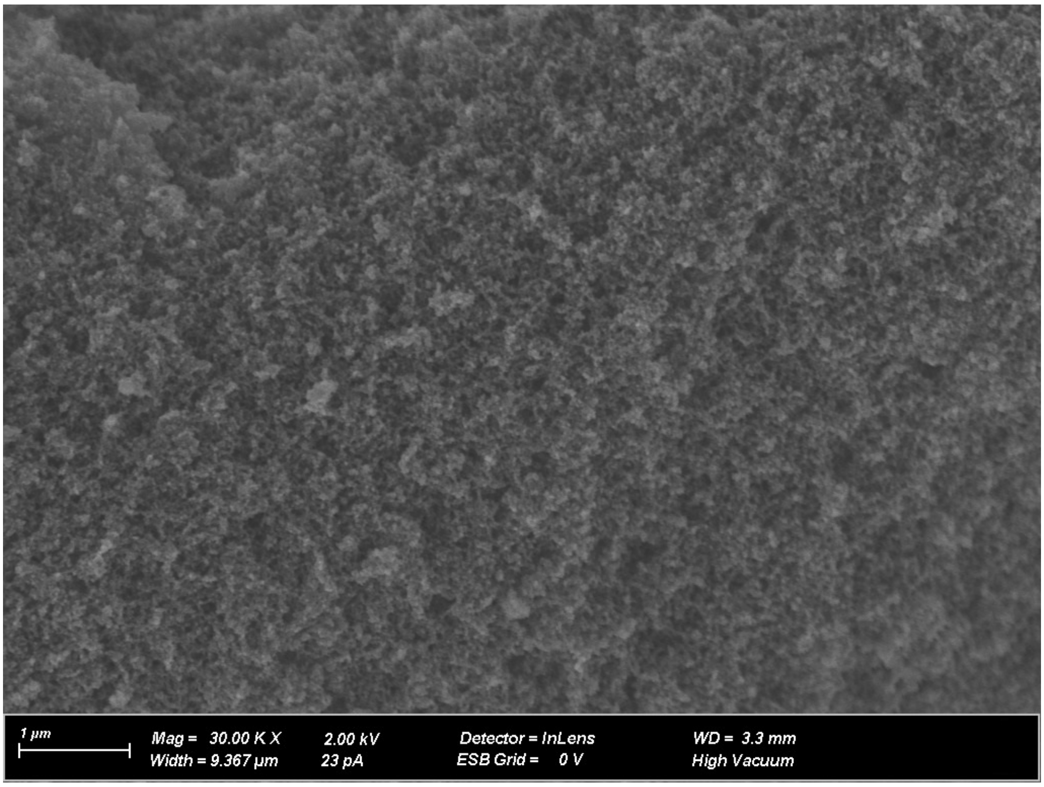

Figure S5: SEM image of Aerogel: 2amino-16C-TMS-3Vol-Aero sample

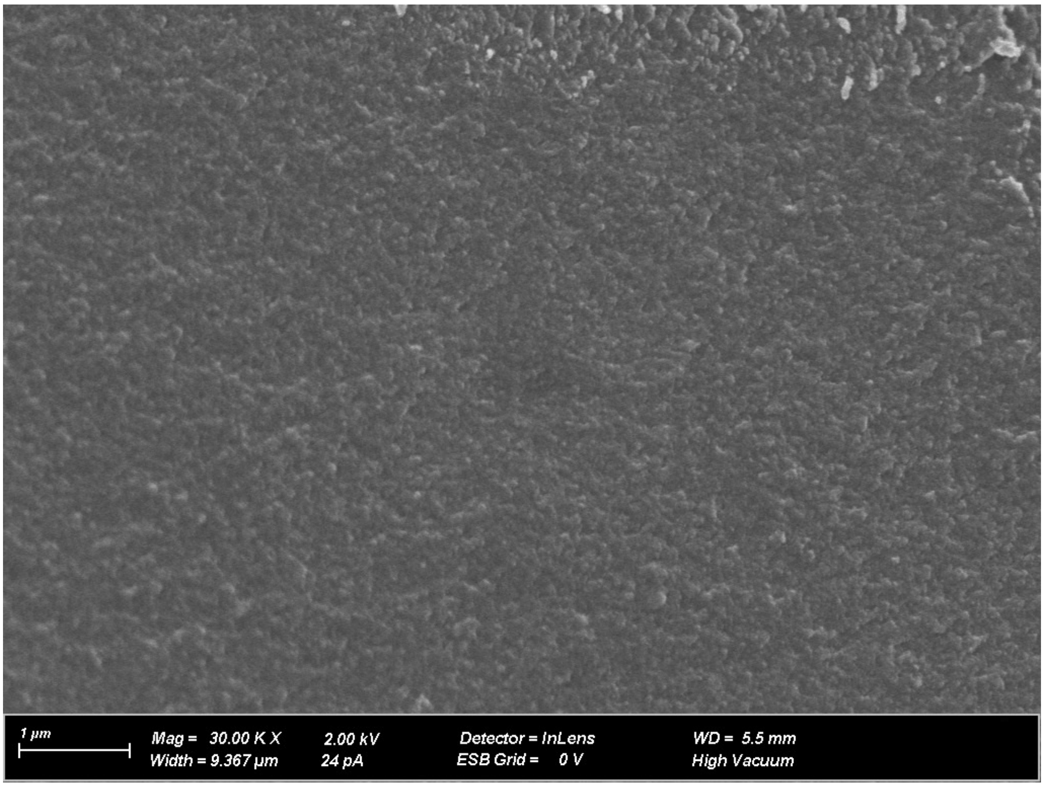

Figure S6: SEM image of Xerogel: 2amino-16C-TMS-3Vol-Xero sample

7 Optical Microscopy

The Silica gel particles were imaged using a Zeiss Imager.M2m Axio microscope (×2.5, ×5 objectives) and with the use of Circular differential interference contrast (C-DIC) for x5 objective. The particle

size distribution (chord length) obtained after sieving the gels is shown in Figure 8.

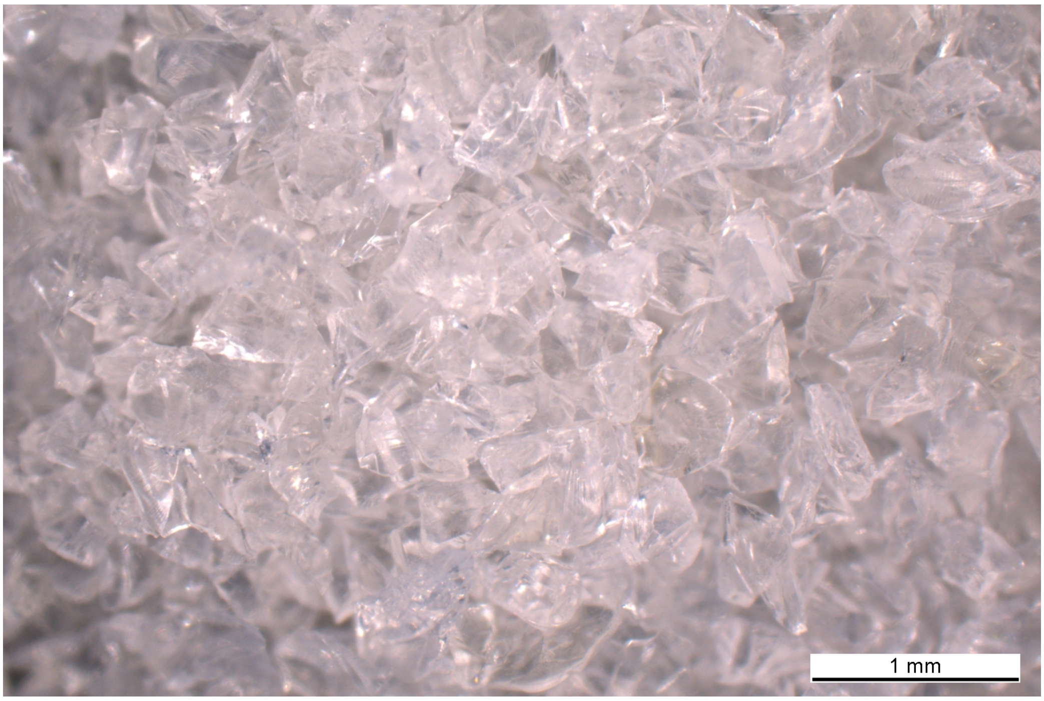

Figure S7: Optical Microscope image of grounded Xerogel: 2amino-Xero. Fraction 0.4 mm for BTA measurements.

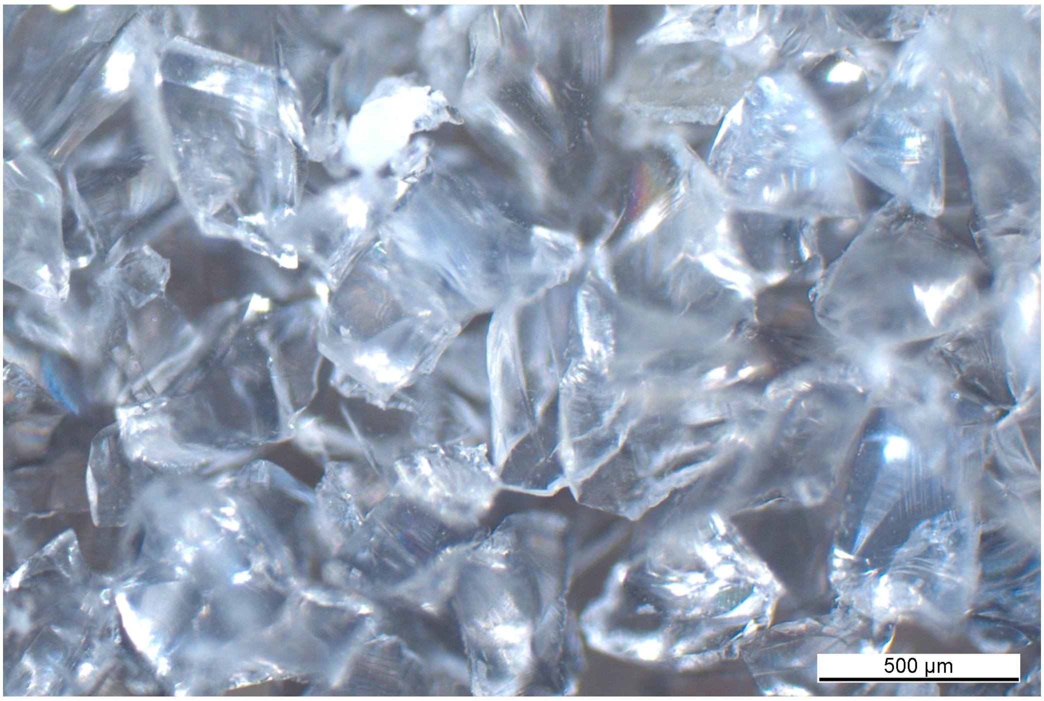

Figure S8: Optical Microscope image of grounded Xerogel: 2amino-Xero. Fraction 0.4 mm for BTA measurements.

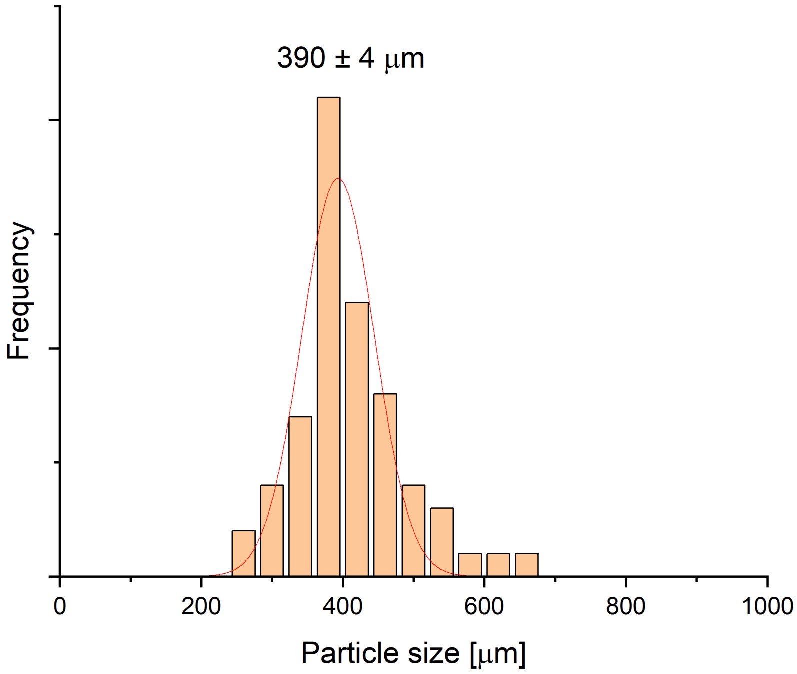

Figure S9: Particle size distribution by number of the sieved 2amino-Xero sample obtained by measuring chord length in random directions from the optical microscopy images.

8 Water Contact Angle

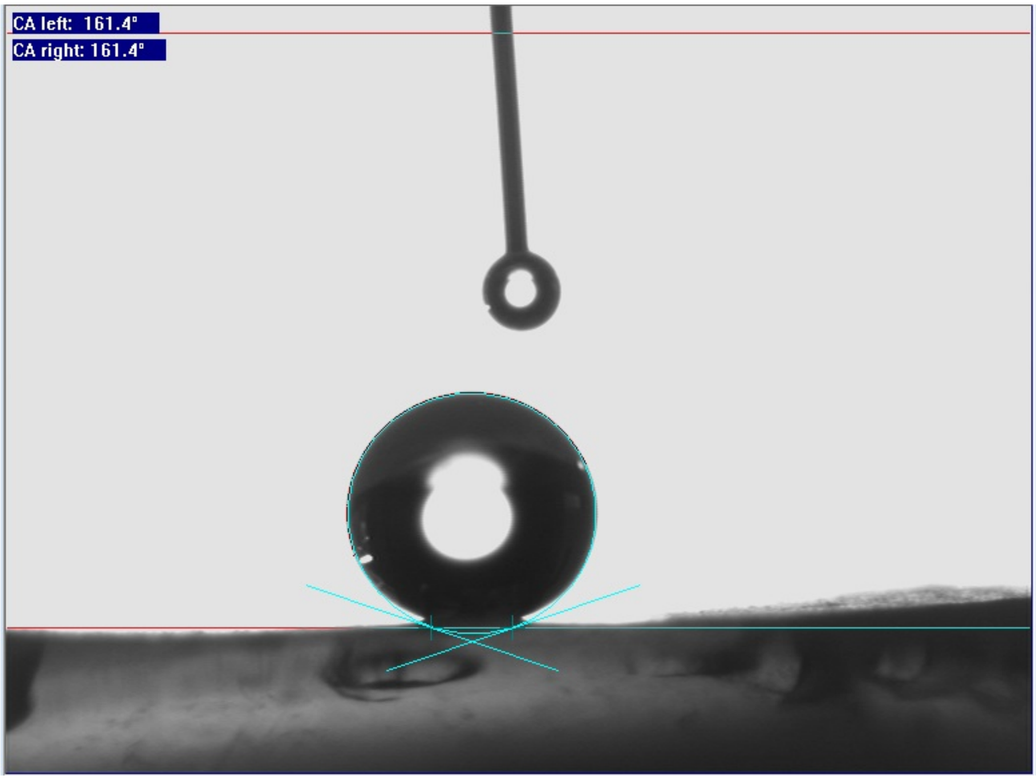

Figure S10: Water contact angle of 160°, measured on the 2amino-16C-TMS-3Vol-Aero, confirming the superhydrophobicity of the sample.

References

[1] Rajendran A. Wilkins N. S. Sawada J. A. "Quantitative Microscale Dynamic Column Breakthrough Apparatus for Measurement of Unary and Binary Adsorption Equilibria on Milligram Quantities of Adsorbents". In: *Industrial and Engineering Chemistry Research* (2022).

[2] Taylor J.R. *An Introduction to Error Analysis*. University Science Books, 1982.

[3] S. Shrinjay et al. "RUPTURA: simulation code for breakthrough, ideal adsorption solution theory computations, and fitting of isotherm models." en. In: *Molecular Simulation* 49.9 (2023), pp. 893–953.

[4] Douglas M. Ruthven. *Principles of adsorption and adsorption processes*. en. New York: Wiley, 1984. ISBN: 978-0-471-86606-0.

[5] H.S. Carslaw and J.C. Jaeger. *Conduction of Heat in Solids*. en. 2nd. Great Britain: Oxford University Press, 1959.

[6] I. Lunati and S. H. Lee. "A Dual-Tube Model for Gas Dynamics in Fractured Nanoporous Shale Formations". en. In: *Journal of Fluid Mechanics* 757 (2014), pp. 943–971. DOI: 10.1017/jfm.2014.519.

[7] P. Goyal, M. J. Purdue, and S. Farooq. "Adsorption and Diffusion of N_2 and CO_2 and Their Mixture on Silica Gel". en. In: *Industrial & Engineering Chemistry Research* 58.42 (2019), pp. 19611–19622. DOI: 10.1021/acs.iecr.9b02685.

[8] J.-T. Anyanwu, Y. Wang, and R. T. Yang. "Amine-Grafted Silica Gels for CO_2 Capture Including Direct Air Capture". en. In: *Industrial & Engineering Chemistry Research* 59.15 (2020), pp. 7072–7079. DOI: 10.1021/acs.iecr.9b05228.

[9] M. Ostwal et al. "3-Aminopropyltriethoxysilane Functionalized Inorganic Membranes for High Temperature CO_2/N_2 Separation". en. In: *Journal of Membrane Science* 369.1-2 (2011), pp. 139–147. DOI: 10.1016/j.memsci.2010.11.053.

[10] K. Wörmeyer and I. Smirnova. "Breakthrough Measurements of CO_2 through Aminofunctionalised Aerogel Adsorbent at Low Partial Pressure: Experiment and Modeling". en. In: *Microporous and Mesoporous Materials* 184 (2014), pp. 61–69. DOI: 10.1016/j.micromeso.2013.09.032.
